# Water soaking in strawberry (*Fragaria* × *ananassa*) has a polygenic background and is strongly influenced by environmental factors

**DOI:** 10.1186/s12870-026-08778-2

**Published:** 2026-05-11

**Authors:** Diana Seidler, Moritz Knoche, Klaus Olbricht, Henryk Flachowsky, Ofere Francis Emeriewen

**Affiliations:** 1 Gottfried Wilhelm Leibniz University Hannover, Institute for Horticultural Production Systems, Hannover, Germany; 2 Julius Kühn Institute, Federal Research Centre for Cultivated Plants, Institute for Breeding Research On Fruit Crops, Dresden, Germany; 3 Hansabred GmbH & Co. KG, Dresden, Germany

**Keywords:** Water soaking, *Fragaria* × *ananassa*, Genetic linkage mapping, QTL analysis, Broad-sense heritability

## Abstract

**Background:**

Water soaking (WS) is an economically important surface disorder in open-field production of strawberry (*Fragaria* × *ananassa*). The disorder, which impairs fruit quality and quantity, develops after exposure of maturing fruit to rain or high concentrations of water vapor. The susceptibility to WS differs among genotypes, suggesting a genetic component, with earlier studies establishing a close positive relationship between susceptibility to WS and the water permeance of the fruit skin. However, until now, no published studies on the underlying genetics of WS exist. Here, we aimed to identify genetic regions associated with WS and fruit skin permeability using a genetic mapping approach.

**Results:**

A biparental F_1_ population was established by crossing two genotypes (201409 × 210706) exhibiting contrasting susceptibility to WS. The trueness-to-type of the progenies was confirmed by genetic fingerprint analysis with molecular markers. WS and permeance for water uptake were phenotyped over two seasons under two different growing conditions using laboratory-based incubation assays. Furthermore, the population was genotyped with the Axiom™ Strawberry FanaSNP 50K Genotyping Array to facilitate genetic mapping and QTL analyses. WS and skin permeance showed a clear segregation and were closely related. Multiple QTL regions were identified for WS and permeance, with one accounting for 16.7% to 28.6% of the phenotypic variation.

**Conclusion:**

The detection of several QTLs and the moderate broad-sense heritability of 0.49 suggest a polygenic background of WS. These findings provide the first steps towards understanding the genetic background of this trait, which will allow for developing tolerant cultivars for open-field production.

**Supplementary Information:**

The online version contains supplementary material available at 10.1186/s12870-026-08778-2.

## Introduction

Water soaking (WS) is an economically important surface disorder of cultivated strawberries (*Fragaria* × *ananassa* Duchesne) produced in the open-field. The disorder emerges when the surface of developing and mature fruit comes into contact with water or high concentrations of water vapor over extended periods of time [27, 29, 30]. Typical symptoms of WS are pale and translucent patches of fruit skin [29, 30]⁠ that increase in area with time [30]⁠. WS reduces attractiveness to consumers, decreases shelf life, and increases harvesting and marketing costs [27]. The physiology of WS in strawberries has been unraveled in recent years [30–36]. The mechanism of WS resembles that of rain cracking of sweet cherry fruits [67, 76]. The formation of microscopic cracks in the cuticle membrane, also known as microcracking, is the first visible step in WS of strawberry [33] ⁠and rain cracking of sweet cherries [47, 57]. Microcracking results from excessive strain of the cuticle due to the downregulation of genes involved in cuticle synthesis and deposition during early development. Subsequent fruit growth distributes an essentially constant amount of cuticle over an increasing surface area, resulting in the thinning of the cuticle, increased strain of the cuticle and the formation of microcracks [48, 70]. Microcracking of the strained cuticle is aggravated by surface water and high humidity [33, 47]. Microcracks impair the barrier function of the cuticle, resulting in increased permeability for water uptake and transpiration. Water uptake that now bypasses the cuticle barrier causes bursting of epidermal and parenchyma cells and the release of organic acids from the vacuole into the cell wall space. Organic acids, in turn, increase the permeability of membranes of neighboring cells, causing further leakage. This chain reaction results in the spreading of WS across the fruit surface [30].

Countermeasures to reduce or prevent WS are limited. Shifting production from the open field to protected cultivation reduces surface wetness duration and hence, WS and fruit rots [54]. However, protected cultivation is associated with higher energy and considerable investment costs [46, 53]. Further, the application of Calcium ions (Ca) reduces WS [31, 38]. Unfortunately, the xylem becomes dysfunctional during fruit development, resulting in reduced Ca import and decreased Ca/dry mass ratios [37, 77]. Thus, only spray application of Ca salts increases fruit Ca [39] and hence, reduces but does not eliminate WS. Strawberry cultivars differ in susceptibility to WS, but there is no information on the genetic background of these differences [27–29, 36]. A better understanding is needed to systematically exploit such differences in breeding less susceptible cultivars suitable for open-field cultivation.

Modern breeding techniques like marker-assisted selection (MAS) [18] play important roles in the present and future of plant breeding [1, 49]. Molecular markers, particularly simple sequence repeats (SSRs) and single nucleotide polymorphisms (SNPs), provide powerful tools for analyzing genetic diversity, population structure, linkage mapping, and identification of quantitative trait loci (QTL) [75]. Nevertheless, breeding of *F*. × *ananassa* is challenging due to its complex allo-octoploid genome (2n = 8x = 56), but recent advances, like the publication of the first octoploid reference genome [21] and the development of the Axiom™ Strawberry FanaSNP 50K Genotyping Array [24] enable building up a powerful basis for molecular breeding in this complex species. Although many QTLs have been found for fruit flowering [12, 13, 69], fruit quality [3, 23, 50, 62, 81], various metabolites [5, 55, 68], fruit skin color [11]⁠ or resistance against pathogens [6, 56, 58, 63, 64], no marker-trait association has yet been reported for WS.

The objective of our study is to gain insight into the molecular and genetic basis of WS using a genetic mapping approach. First, a segregating F_1_ population was established by crossing two genotypes showing contrasting susceptibilities to WS. Second, the F_1_ population was genotyped using the Axiom™ Strawberry FanaSNP array and a set of SSR markers, and thereafter, phenotyped for WS and the skin permeance for water uptake (P_f_). Finally, marker-trait associations for WS susceptibility and P_f_ were identified. The results of this study contribute to a better understanding of the genetic basis of WS and provide a basis for the breeding of WS-tolerant strawberry cultivars.

## Material and methods

### Plant material and growth conditions

Plants of *Fragaria* × *ananassa* were provided and raised by Hansabred GmbH & Co. KG in Dresden-Weixdorf, Germany (51°09′09.4"N 13°47′58.7"E). In 2023, the F_1_ population (*n* = 263) was established from the biparental cross between the breeding clones 201409 (Susette × Lola) and 210706 (190222 × pollen mixture of breeding clones).

Cross-breeding was performed under greenhouse conditions using a forcing culture (day/night temperature of 20/15 °C, long-day artificial lighting of 16 h). The male parent was cultivated first; pollen was collected and stored in an exsiccator under hygroscopic conditions in the refrigerator. The female parent was cultivated approximately three weeks later to ensure that viable pollen was available at the pistil ripening stage. Emasculation of the flowers was performed at the balloon stage to avoid self-pollination with ripe pollen. During both the pollination period and pollen harvest, the inflorescences were covered with a plastic bag to prevent open pollination.

In 2024, the F_1_ seedlings were raised in pots filled with substrate (PRO berry red PE20; Kekkilä-Brill, Georgsdorf, Germany) under greenhouse conditions (heating at temperatures below 4 °C, venting at temperatures above 18 °C). Irrigation and fertilization were provided as needed using 0.2% mineral NPK fertilizer (Peters® Excel CalMag Grower; ICL Europe B.V. Growing Solutions, Nordhorn, Germany). After the fruiting season, the NPK fertilizer Peters® Excel CalMag Finisher (ICL Europe B.V. Growing Solutions, Nordhorn, Germany) was used at the same concentration. In addition, foliar fertilisers were applied as sprays several times during the fruiting period (0.3% WUXAL® Calcium; Hauert MANNA Düngerwerke, Nürnberg, Germany and 0.2% Lebosol® Kalium Plus; Lebosol® Dünger, Elmstein, Germany). After the harvest season, the F_1_ population and both parents were vegetatively propagated by runners. For genotypes where insufficient runners were produced, the mother plants were kept for the following phenotyping season. From November 2024 until May 2025, the clones were frozen as tray plants at −2 °C, then thawed, potted, and placed into a high tunnel table-top system outside under a rain cover. Irrigation and fertilization were performed using a drip irrigation system (0.12–0.16% mineral NPK fertilizer Peters® Excel CalMag Grower; ICL Europe B.V. Growing Solutions, Nordhorn, Germany). All plants were cultivated according to current IPM regulations and practices [22].

### Phenotyping

Phenotyping for susceptibility to WS (ordinal, qualitative method) and fruit skin permeance P_f_ (metric, quantitative method) was performed in two consecutive seasons. In each season, a minimum of eight to a maximum of 16 ripe fruits, free of visual damage, were harvested while avoiding direct contact with the fruit skin. Fruits were placed into a soft foam tray, and the peduncle end was sealed with silicone rubber (Silicone RTV; Dow Toray Co., Ltd., Tokyo, Japan) to prevent water loss or uptake. Because of the shortage of fruits from the seedlings in the 2024 season, fruits were sampled regardless of position within the inflorescence. In 2025, secondary and tertiary fruits were sampled; the large primary fruits were avoided.

For phenotyping of WS, the laboratory assay by Hurtado and Knoche [30] was used. Briefly, fruits were incubated individually in deionized water for 4 h at room temperature. A soft foam plug ensured submersion of the whole fruit. After 4 h, fruits were removed and carefully blotted with soft tissue paper (Kimtech® Science Task Wipes; Kimberly-Clark Professional™, Koblenz, Germany). The severity of WS symptoms was assessed by visual rating using the following rating scale: score 0, no WS; score 1, < 10% of the fruit surface area water-soaked; score 2, 10–35%; score 3, 36–60%; score 4, > 60% [30].

The permeance for water uptake (P_f_, m s^−1^) was determined on an individual fruit basis using Eq. (1) by Hurtado et al. [36]. Fruit with pedicel end sealed using silicone rubber were weighed, incubated individually in deionized water for 4 h, removed from the solution and blotted and reweighed.

1 $${P}_{f} = \frac{{F}_{f}}{{A}_{fruit}\cdot \Delta \Psi }\cdot \frac{R\cdot T}{\rho \cdot \overline{{V}_{W}}}$$

The rate of uptake (F_f_; mg h^−1^) was calculated from the increase in fruit mass during incubation. This calculation assumes the rate of water uptake to remain constant. The latter part of the Eq. (1) represents the universal gas constant (R, m^3^ Pa mol^−1^ K^−1^), the absolute temperature (T, K), the density (ρ, kg m^−3^), and the molar volume of water (V_w_, m^3^ mol^−1^) [36]. The fruit surface (A_fruit_, cm^2^) was calculated from fruit mass using Eq. (2) by Hurtado et al. [36].

2 $${A}_{fruit}\text{ = }5.0756\cdot {(mass)}^{0.6547}$$

The difference between the water potential of the fruit (Ψ_fruit_, MPa) and the water potential of the incubation solution (here Ψ = 0 MPa) is represented by ΔΨ. Since the turgor of strawberry is negligible [35]⁠, Ψ_fruit_ effectively equals the osmotic potential of the expressed juice (Ψ_π_, MPa) [36]⁠. The latter is closely related to the content of total soluble solids (TSS, °Brix) and was estimated using Eq. (3) by Straube et al. [70]. The TSS content was determined using a hand-held refractometer (PAL 1, Atago Co., Ltd. Tokyo, Japan) on juice extracted using a garlic press.

3 $${\Psi }_{\pi } = 0.3292 - 0.0421\cdot TSS - 0.0088\cdot {TSS}^{2} + 0.0002\cdot {TSS}^{3}$$

For both WS and P_f_ traits, the mean values were calculated and used for statistical analyses.

### Statistical analysis and broad-sense heritability H^2^

Statistical analyses were conducted in R version 4.4.1 [60] using the RStudio integrated development environment version 2025.09.0 [59]⁠. Because of the ordinal character of the WS trait, correlation analyses were conducted after Spearman by using the *psych* package version 2.6.1 [61]. Similarly, comparisons of phenotypic characteristics of genotypes were made using the R package *rstatix* version 0.7.2 [44] and non-parametric Kruskal–Wallis rank sum tests and post-hoc Dunns tests. Pairwise comparisons between values of both seasons were made with a non-parametric Wilcoxon test using the *stats* package version 4.4.1 [60].

The broad-sense heritability H^2^ was computed with the *lme4breeding* package version 1.0.80 [7, 19] for every single season and for both seasons together. Variance components were derived from a linear model with genotype × environment interaction as random effects. For each season, H^2^ was calculated as H^2^ = V_G_/(V_G_ + V_E_), where V_G_ was the variance among genotype means, and V_E_ was the residual variance within the genotypes [19].

### Genotyping

#### Single nucleotide polymorphism (SNP) marker analysis

Fresh unfolded leaves of each F_1_ genotype and the parental genotypes were selected, cut into ten small pieces (3–4 mm) and placed into microwell plates, and freeze-dried. The DNA extraction and genotyping were performed by SGS Institute Fresenius GmbH (Section Trait Genetics, Gatersleben, Germany) with the Axiom™ Strawberry FanaSNP 50K Genotyping Array, Thermo Fisher Scientific Inc. [24]. Marker sequences were blasted against the reference genome of *F.* × *ananassa* cv. ˈCamarosaˈ v.1.0 assembly [21] to identify the chromosome and physical position information. The results of SNP data analysis and remaining plant DNA (concentration range 20–50 ng) were transferred to the Julius Kuehn Institute, Federal Research Centre for Cultivated Plants in Dresden-Pillnitz, Germany, where further molecular analyses were performed. The DNA concentration of the samples was adjusted to 1–10 ng/µl for PCR analyses.

#### Microsatellite (SSR) marker analysis and F_1_ population fingerprint

Twenty SSR primer pairs (Table S1, Supplementary Information), labelled with different fluorescent dyes (Microsynth ecogenics GmbH, Balgach, Switzerland), were applied to both parents and the population to serve as anchor markers. Multiplex-PCR (MP) was performed with a commercial kit (Type-it Microsatellite PCR Kit; QIAGEN®, Düsseldorf, Germany). One microliter of DNA sample (1–10 ng/µl) was mixed with 1.15 µl RNAse-free water, 3.0 µl Multiplex PCR Mastermix (2 x), 0.6 µl Q-Solution® (5 x), and 0.25 µl primer mix (1 µM). PCR conditions were 95 °C for 1 min, followed by 35 cycles of 95 °C for 1 min, 56 °C for 1 min, 72 °C for 1 min, and an extension at 60 °C for 30 min. For fragment analysis with capillary gel electrophoresis, PCR products were diluted with 200 µl of deionized water, and 1 µl of the dilution was mixed in a MicroAmp™ Optical 96-Well reaction plate with 8.95 µl of Hi-Di™ Formamide and 0.05 µl GeneScan™ – 600 LIZ® Size Standard. The samples were denatured for 5 min at 95 °C before analysis on the 3500 Genetic Analyzer. Fragments were visualized with the GeneMapper™ Software. All reagents, the 3500 Genetic Analyzer, and the corresponding software were provided by Applied Biosystems™ (Fisher Scientific GmbH, Schwerte, Germany).

To ascertain the genetic composition of the F_1_ population, the allele patterns of all F_1_ genotypes, resulting from SSR marker analysis, were compared to the parental genotypes. Genotypes with exclusively maternal alleles were defined as selfers, and genotypes with foreign alleles, which could not be attributed to the parental genotypes 201409 or 210706, were classified as outcrossers. Due to the octoploid genome of *F*. × *ananassa*, the R package *polysat* [15, 16] was used for the calculation of allele frequency, expected heterozygosity (H_exp_) and polymorphism information content (PIC). The pairwise genetic distance matrix between individuals were estimated with the default Bruvo.distance measurement.

#### Genetic linkage mapping

Linkage maps of each parent using the SNP and SSR markers were constructed with JoinMap® 4.0 [73]. SNP Markers were labelled with the number of chromosomes and subgenomes according to the *F.* × *ananassa* cv. ˈCamarosaˈ v.1.0 assembly reference genome [21]. Only SNP markers of the categories PolyHighResolution (n clust = 3, FLD > 5, MAF > 0.05) and NoMinorHom (n clust = 2, FLD > 5, MAF > 0.05) were selected. For each parental genotype, markers with missing data, indels and homozygotes were deleted, and nucleotide combinations of the remaining SNP markers were transformed into segregation types < lm × ll >, < hk × hk > or < nn × np >, according to the JoinMap® 4.0 software instructions for cross populations CP. Additionally, selected polymorphic SSR markers were included in the mapping process. Genotypes identified as selfers or outcrossers were excluded from the mapping analysis. Only markers that segregated in both parents (segregation type < ef × eg >, < ab × cd > and < hk × hk >) were used. Additionally, for the maternal map, markers that segregated in only the maternal parent (segregation type < lm × ll >) were included. Similarly, markers that segregated in only the paternal parent (segregation type < nn × np >) were added to the paternal map. Markers that included more than 5% of genotypes with missing data were removed, as well as markers with a highly significant segregation distortion (SD) of X^2^ > 40 and a similarity of 1.0. Grouping was carried out with default settings of JoinMap® 4.0 and an independence Logarithm of the odds score (LOD) between 8 and 15. Regression mapping with three rounds was performed with Kosambi’s function. For calculations, the function for the CP population type was used. Groups including fewer than 50 markers were not mapped. Markers, which differed from chromosome affiliation within one group, were excluded. In case of insufficient linkage phase determination, ungrouped loci were excluded until the JoinMap® 4 software was able to proceed. If one group consisted of multiple sets of markers from the same chromosome but different subgenomes, these markers were mapped separately. Linkage groups were named according to the recent *F.* × *ananassa* nomenclature [26], and the quality of mapping was verified by comparison of the map distance and physical position of the markers.

### QTL analysis

The respective genetic map data and the mean values from phenotyping the F_1_ population for each season for WS and P_f_ were used for QTL analyses, which were performed with MapQTL® 5.0 [72]. Traits were log-transformed if skewness and kurtosis were below −2 and above 2 (based on session information of MapQTL® 5.0). The level of significance between marker-phenotype associations was estimated with the Kruskal–Wallis rank-sum test (KW). Interval mapping (IM) was conducted with default settings, and the genome-wide LOD threshold (LOD_GW_) and the chromosome-wide LOD thresholds (LOD_CW_) for each linkage group were calculated with a 1000-iteration permutation test (PT) for each trait at a *p*-value < 0.05. Regions with significantly linked markers above the LOD_CW_ threshold were considered as QTLs. The maximum LOD scores (LOD_max_) and the LOD-1 (LOD_max_−1) and LOD-2 intervals (LOD_max_−2) were calculated to determine the putative position of the QTL. The visualisation of the genetic maps and QTL results was performed with MapChart 2.32 [74] and modified in Inkscape 1.3.2 [40]. For each identified QTL, the number of genes within the area above LOD_CW_ was determined with the JBrowse tool of the GDR database [42], using the reference genome of *F.* × *ananassa* ˈCamarosaˈ Genome Assembly v1.0.a2 [21, 51] ⁠ and ˈRoyal Royceˈ Genome v1.0 [25]. For a selection of QTLs, genes underlying the marker with the highest LOD score were compared to the GDR database.

## Results

### SSR marker analysis revealed two subpopulations

Ninety-five percent of the 20 SSR markers led to sufficient amplification (Table 1). The only exception was marker CFVCT016, where no alleles could be detected. The number of alleles per locus ranged from one to six, with an average number of 2.9 alleles. Most of the markers were bi- (32%) or multiallelic (58%), but two of them were single-allelic (EMFv030 and EMFv001). In total, 55 alleles were determined, and 63% of them were polymorphic. In the case of marker ChFaM036, the missing allele information of the paternal genotype 210706 was treated as a null allele. The lowest expected heterozygosity was calculated for the single-allele markers EMFv030 and EMFv001 (H_exp_ = 0.00). For biallelic and multiallelic markers, H_exp_ ranged from 0.22 (UFFa_09E12) to 0.83 (UDF-004). The polymorphic content was highest for markers UDF-004 and UDF-009 (PIC = 0.80) and lowest for EMFv030 and EMFv001 (PIC = 0.00).

**Table 1:** SSR marker analysis of the F_1_ 201409 × 210706 cross population with number of alleles (n_a_), allele size (bp) and relative allele frequency, expected heterozygosity (H_exp_) and polymorphic information content (PIC), based on PCR fragment analysis and the *polysat* analysis [15, 16]. Markers which were polymorphic (_p_) were used for genetic mapping

		**Allele size bp (allele frequency)**							
**Marker**	**n**_**a**_	**Allele 1**	**Allele 2**	**Allele 3**	**Allele 4**	**Allele 5**	**Allele 6**	**H**_**exp**_	**PIC**
UFFa_13C07_p_	2	164_ m_ (0.61)	173_p_ (0.39)					0.48	0.36
CFVCT028_p_	2	141_ m_ (0.83)	143_p_ (0.17) ♂					0.28	0.24
UDF-001_p_	4	162_p_ (0.17) ♂	165_p_ (0.40) ♀	167_p_ (0.11) ♂	169_p_ (0.33) ♀			0.69	0.64
UFFa_11G07_p_	3	172_p_ (0.39)	174_p_ (0.13) ♂	182_ m_ (0.48)				0.60	0.52
UAFv7344_p_	3	206_p_ (0.28)	207_p_ (0.11)	209_ m_ (0.60)				0.54	0.47
SF-A01_m_	3	171_ m_ (0.50)	174 (0.002)	175_ m_ (0.50)				0.50	0.38
UFFa_09E12_p_	2	187_p_ (0.12) ♂	197_ m_ (0.88)					0.22	0.19
EMFv030_m_	1	192_ m_ (1.00)						0.00	0.00
ChFaM036_p_	3	- (0.004) ♂	213_p_ (0.51) ♀	220_p_ (0.49) ♀				0.50	0.38
UDF-009_p_	6	144_p_ (0.20)	145_p_ (0.18)	148_p_ (0.07) ♂	163_p_ (0.16) ♀	171_p_ (0.20)	181_p_ (0.20)	0.82	0.80
UDF-004_p_	6	131_ m_ (0.21)	133_ m_ (0.19)	135_p_ (0.17)	139_p_ (0.12) ♀	141_ m_ (0.18)	154_p_ (0.14)	0.83	0.80
EMFv017_p_	2	199_p_ (0.29) ♀	207_ m_ (0.71)					0.41	0.33
UFFa_01D03_m_	3	240_ m_ (0.50)	243_ m_ (0.50)	246 (0.01)				0.51	0.39
UAFv8316_m_	3	234_ m_ (0.33)	236_ m_ (0.33)	238_ m_ (0.33)				0.67	0.59
MCAD_FAC_001_p_	3	189_p_ (0.15) ♂	191_p_ (0.12) ♂	202_ m_ (0.73) ♀				0.43	0.39
ChFaM138_m_	2	311_ m_ (0.56)	314_ m_ (0.44)					0.49	0.37
EMFv001_m_	1	230_ m_ (1.00)						0.00	0.00
UFFa_03C04_p_	4	240_ m_ (0.31)	246_ m_ (0.30)	249_p_ (0.21) ♀	252_p_ (0.18) ♀			0.74	0.69
ChFaM078_p_	2	252_p_ (0.61)	255_p_ (0.39)					0.48	0.36

*m* monomorphic, *p* polymorphic; *♀* alleles, inherited only from 201409, *♂* alleles, inherited only from 210706

SSR genotyping revealed that 44.1% of the F_1_ population were selfers since they had exclusively maternal allele combinations without any paternal alleles. Some genotypes revealed allele combinations, which were interpreted as implausible (1.5%). The analysis of the markers UFFa_01D03 and SF-A01 revealed six genotypes with foreign alleles, which were classified as outcrossers (2.3%). Principal component analysis (PCA) and frequency distribution of individual genetic distances showed two subpopulations, each containing one parent (Fig. S1, Supplementary Information). Of 263 progenies of the F_1_ population, only 52% revealed truly biparental allele patterns and were thus used for phenotyping and genetic mapping (*n* = 136 genotypes).

### Phenotyping over two seasons under different conditions

**Fig. 1:**
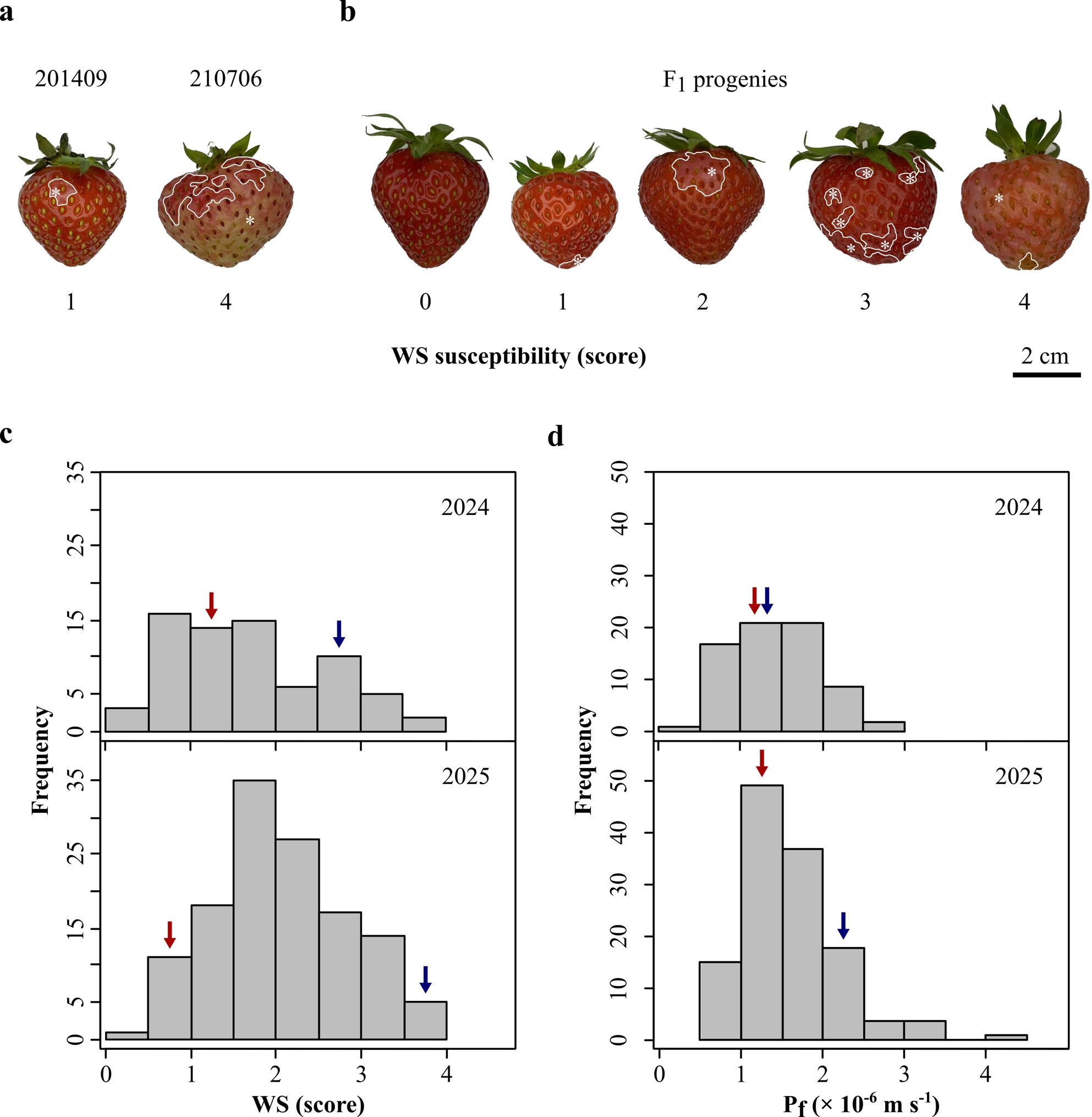
Water soaking (WS) and skin permeance in water uptake (P_f_) in the parental genotypes and the segregating F_1_ population. **a** WS symptoms of parental genotypes 201409 (maternal) and 210706 (paternal). **b** Selected F_1_ progenies that represented the rating scheme used for assessing WS. WS was indexed after 4 h incubation in deionized water using a 5-point rating scale: score 0, no WS; score 1, < 10% of the fruit surface area water-soaked; score 2, 10-35%; score 3, 36-60%; score 4, > 60% [30]. Asterisks indicated the water-soaked area. **c** Distribution of WS and **d** P_f_ in a *F*. × ananassa F_1_ 201409 × 210706 cross population over two seasons (2024, n = 71, 2025, n = 128). Skin permeance P_f_ was calculated as described in Hurtado et al. [36]. Values of parental genotypes were represented by arrows (red, 201409; blue, 210706)

The number of progenies used for phenotypic evaluation was 71 in 2024 and 128 in 2025. The parents 201409 and 210706 differed significantly in susceptibility to WS (Fig. 1a, Table 2) with a low overall WS score of 0.97 ± 0.12 for 201409 and a high overall WS score of 2.87 ± 0.19 for 210706 (significant in both years at *p* < 0.001). In 2025, WS was highly variable for 201409 (coefficients of variation CV of 82%) compared to 210706, which had a CV in 2024 of 41%. The F_1_ population clearly segregated for WS (Fig. 1b and c, Table 2) with an overall mean score of 2.00 ± 0.07.

**Table 2:** Water soaking (WS) and fruit skin permeance in water uptake (P_f_) phenotyped in two seasons in the F_1_ cross population and their parental genotypes 201409 and 210706 (number N of observed fruits of the parental genotypes or genotypes of the F_1_ population, mean, standard error SE; median, minimum, maximum, coefficient of variation CV%). WS was indexed after 4 h incubation in deionized water using a 5-point rating scale: score 0, no WS; score 1, < 10% of the fruit surface area water-soaked; score 2, 10–35%; score 3, 36–60%; score 4, > 60% [30] and P_f_ was calculated as described in Hurtado et al. [36]

**WS (score)**							
**Genotype/population**	**N**	**Year**	**Mean ± SE**	**Median**	**Min**	**Max**	**CV%**
201409	20	2024	1.00 ± 0.15	1.00	0.00	3.00	64.89
	10	2025	0.90 ± 0.23^ ns^	1.00	0.00	2.00	81.98
	30	total	0.97 ± 0.12	1.00	0.00	3.00	69.17
210706	20	2024	2.55 ± 0.23	3.00	1.00	4.00	41.18
	10	2025	3.50 ± 0.22*	4.00	2.00	4.00	20.20
	30	total	2.87 ± 0.19	3.00	1.00	4.00	36.34
F_1_ population	71	2024	1.79 ± 0.10	1.70	0.30	3.60	47.66
	128	2025	2.08 ± 0.07*	2.00	0.30	3.80	37.94
	130	total	2.01 ± 0.07	2.00	0.30	3.70	37.29

**P**_**f**_** (× 10**^**–6**^** m s**^**−1**^**)**							
**Genotype/population**	**N**	**Year**	**Mean ± SE**	**Median**	**Min**	**Max**	**CV%**
201409	20	2024	1.09 ± 0.12	0.99	0.49	2.20	48.81
	10	2025	1.25 ± 0.28^ ns^	0.88	0.54	3.11	70.14
	30	total	1.14 ± 0.12	0.94	0.49	3.11	57.48
210706	20	2024	1.09 ± 0.09	0.89	0.58	1.94	38.03
	10	2025	2.11 ± 0.22***	1.92	0.80	3.22	32.79
	30	total	1.43 ± 0.13	1.31	0.58	3.22	49.41
F_1_ population	71	2024	1.43 ± 0.06	1.45	0.48	2.77	37.42
	128	2025	1.61 ± 0.05 ns	1.49	0.54	4.15	37.54
	130	total	1.57 ± 0.05	1.44	0.58	4.15	36.08

Asterisks indicate significant differences of the means between both seasons (pairwise wilcoxon test, *p* < 0.05, *; *p* < 0.001, ***; *p* > 0.05, *ns* not significant)

The overall P_f_ was higher for the more WS-susceptible parent 210706 (P_f_ = 1.43 ± 0.13 ∙ 10^–6^ m s^−1^, Table 2) than for the less WS-susceptible parent 201409 (P_f_ = 1.14 ± 0.12 ∙ 10^–6^ m s^−1^). Differences in skin permeances of the two parents were significant only in 2025 (*p* < 0.05), but not in 2024 (*p* = 0.6395). Similar to WS, the F_1_ population segregated in P_f_ (Fig. 1d, Table 2). Interestingly, the mean permeance of the progeny was higher than that of either parental genotype (P_f_ = 1.57 ± 0.05 ∙ 10^–6^ m s^−1^). Comparing the 2024 and 2025 seasons revealed no significant differences for WS and P_f_ for 201409 (*p* > 0.05), whereas for 210706 and the F_1_ population, mean WS and P_f_ were significantly higher in 2025 than in 2024 (*p* < 0.01). Correlation analysis of the annual WS and P_f_ means of the F_1_ population revealed highly significant coefficients of correlation (for WS 2024/2025 *r* = 0.52***; for P_f_ 2024/2025 *r* = 0.58***, Fig. S2, Supplementary Information).

Analysis of the frequency distributions of WS score and P_f_ estimates generally revealed normal distribution (Fig. 1c and d). Only in 2025 was the distribution of P_f_ skewed towards higher permeances due to a higher frequency of outliers. Correlation analysis revealed close positive relationships between WS and P_f_ (for 2024 of *r* = 0.83***, and for 2025 *r* = 0.79***, Fig. S2, Supplementary Information). The overall broad-sense heritability H^2^, including genotype × environment interaction, was 0.49 for WS (Table 3; H^2^ _2024_ = 0.39 and H^2^ _2025_ = 0.33). and 0.57 for P_f_, (Table 3; H^2^ _2024_ = 0.44 and H^2^ _2025_ = 0.46). The variance among genotype means (V_G_) was slightly smaller than the residual variance within the genotypes (V_E_).

**Table 3:** Broad sense heritability H^2^ of water soaking (WS) and fruit skin permeance in water uptake (P_f_), phenotyped in two seasons in a *F*. *ananassa* F_1_ 201409 × 210706 cross population (V_G_ = genetic variance or variance among genotype means; V_G×E_ = variance of genotype × environment interaction; V_E_ = residual variance within the genotypes). Computation was carried out with the package *lme4breeding* in R version 4.4.1 [7, 19]

Trait	Year	H^2^	V_G_	V_G×E_	V_E_
WS	2024	0.389	0.629	-	0.987
	2025	0.332	0.544	-	0.986
	total	0.489	0.327	0.243	0.883
P_f_	2024	0.441	2.529 · 10^–13^	-	3.212 · 10^–13^
	2025	0.456	2.801 · 10^–13^	-	4.659 · 10^–13^
	total	0.570	2.055 · 10^–13^	0.952 · 10^–13^	4.282 · 10^–13^

### Genetic linkage mapping

**Fig. 2:**
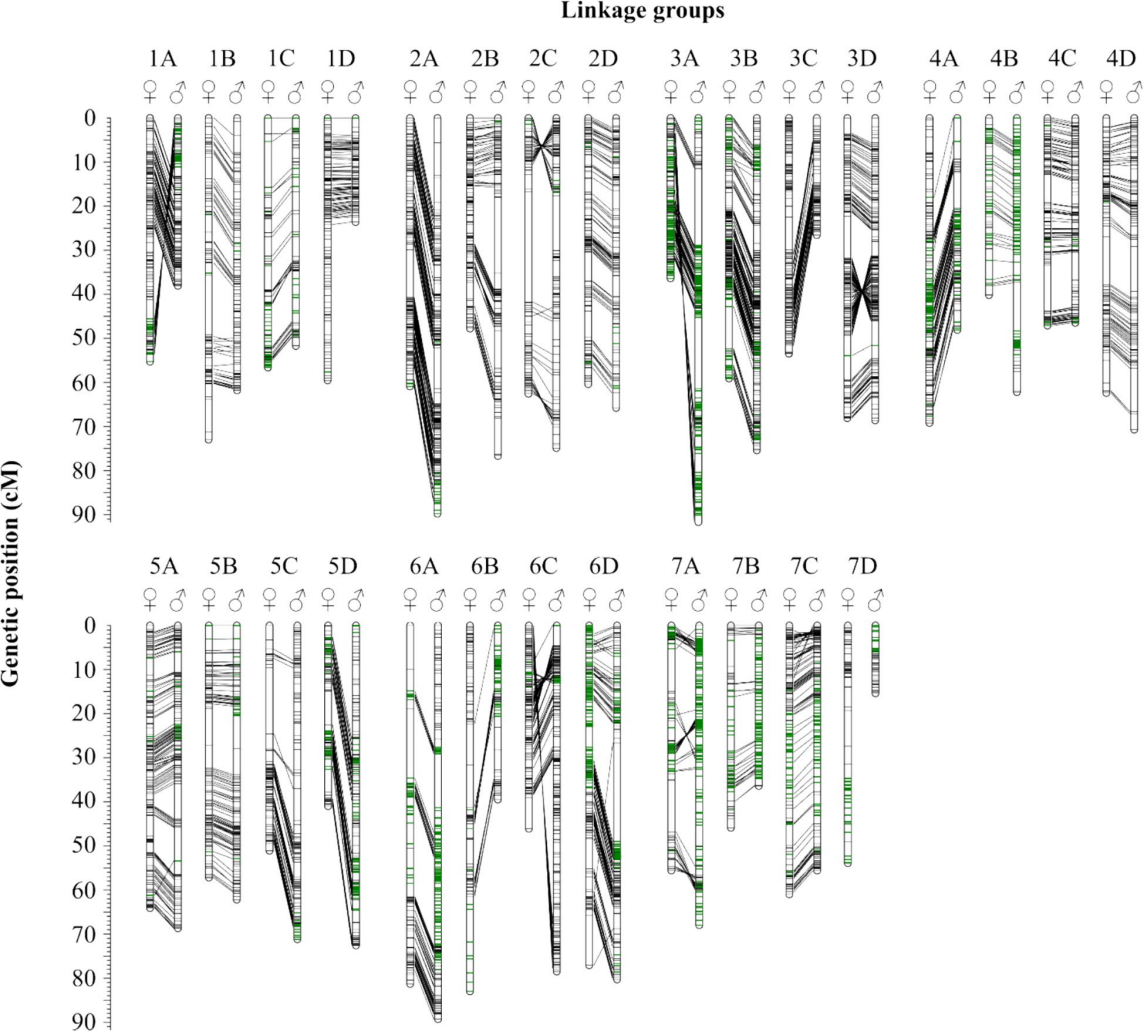
Comparison of both parental maps of *F* × *ananassa* 201409 (maternal, ♀) and 210706 (paternal, ♂) according to the position of identical markers and regions with segregation distortion. Marker, which were mapped on both maps, were connected to each other with black lines. Marker with segregation distortion were marked in green. Linkage groups of the homoeologous groups 1–7 were named according to the recent *F*. × *ananassa* nomenclature A-D [26]

Two parental maps were created, reflecting all seven chromosomes and their subgenomes in a total of 28 linkage groups (Table 4). The map of 201409 with a total of 4,741 markers covered a length of 1,578.3 cM, whereas the map of 210706 included 5,034 markers with a length of 1,710.1 cM. There were remarkable differences in the size of individual maternal and paternal linkage groups (e.g., LG 1D_201409_ = 59.5 cM vs LG 1D_210706_ = 23.6 cM or LG 7D_201409_ = 53.9 cM vs. LG 7D_210706_ = 15.4 cM). Whereas the average marker density across both maps was high, large gaps were found on different linkage groups (Fig. 1; LG 2C, 6A, 7A). Markers with segregation code < hk × hk > were mostly found in the same genetic positions in both maps, but in some linkage groups, these markers were in reverse positions for the other parent map (Fig. 1; LG 1A, 2C, 3D, 6C, 7A). For LG 7D, there was no consistency of markers for both parents. Regions with SD were found on several linkage groups, sometimes only at the proximal or distal region (Fig. 1; LG 1A, 2D, 5C and 7D), but also distributed in a broader range across the linkage groups (Fig. 1; LG 3A, 4B and 7A-C). There was a good collinearity between both parental maps as well as between the genetic and physical positions, although inversion (Fig. 2; LG 1A, 1B, 2C, 3A, 3C, 3D, and 6C) and translocations (Fig. 2; LG 2A, 2D, and 6D) were observed. Examples of poor collinearity are shown in Fig. 2, LG 1B, 4A, 6B, 6D, and 7D.

**Table 4:** Characteristics of the linkage maps of *F.* × *ananassa* 201409 and 201706. For each linkage group (LG), the number of markers, genetic distance and marker density was given. Linkage groups of the homoeologous groups 1–7 were named according to the recent *F.* × *ananassa* nomenclature A-D [26]

	**201409**			**210706**		
**LG**	**n marker**	**Genetic distance (cM)**	**Density (marker/cM)**	**n marker**	**Genetic distance (cM)**	**Density (marker/cM)**
1A	178	55.34	3.22	190	38.11	4.99
1B	133	73.01	1.82	157	61.80	2.54
1C	104	56.63	1.84	88	51.67	1.70
1D	214	59.47	3.60	139	23.56	5.90
2A	218	60.87	3.58	253	89.84	2.82
2B	182	47.82	3.81	177	76.74	2.31
2C	118	62.61	1.88	149	74.88	1.99
2D	196	60.37	3.25	174	65.81	2.64
3A	211	36.41	5.80	221	91.73	2.41
3B	295	59.13	4.99	335	75.38	4.44
3C	202	53.46	3.78	127	26.64	4.77
3D	257	68.10	3.77	277	68.62	4.04
4A	239	69.15	3.46	129	48.14	2.68
4B	75	40.19	1.87	134	62.22	2.15
4C	146	47.11	3.10	157	46.47	3.38
4D	184	62.38	2.95	158	70.73	2.23
5A	226	64.08	3.53	204	68.73	2.97
5B	167	57.24	2.92	203	62.24	3.26
5C	115	51.13	2.25	180	71.16	2.53
5D	120	40.96	2.93	186	72.63	2.56
6A	120	81.34	1.48	227	89.34	2.54
6B	138	82.98	1.66	108	39.47	2.74
6C	208	46.12	4.51	285	78.50	3.63
6D	217	77.14	2.81	218	80.30	2.71
7A	125	55.62	2.25	192	68.00	2.82
7B	91	45.90	1.98	106	36.36	2.92
7C	175	61.00	2.87	186	55.62	3.34
7D	87	53.87	1.61	74	15.39	4.81
Total	4741	1578.29	2.98	5034	1710.07	3.14

**Fig. 3:**
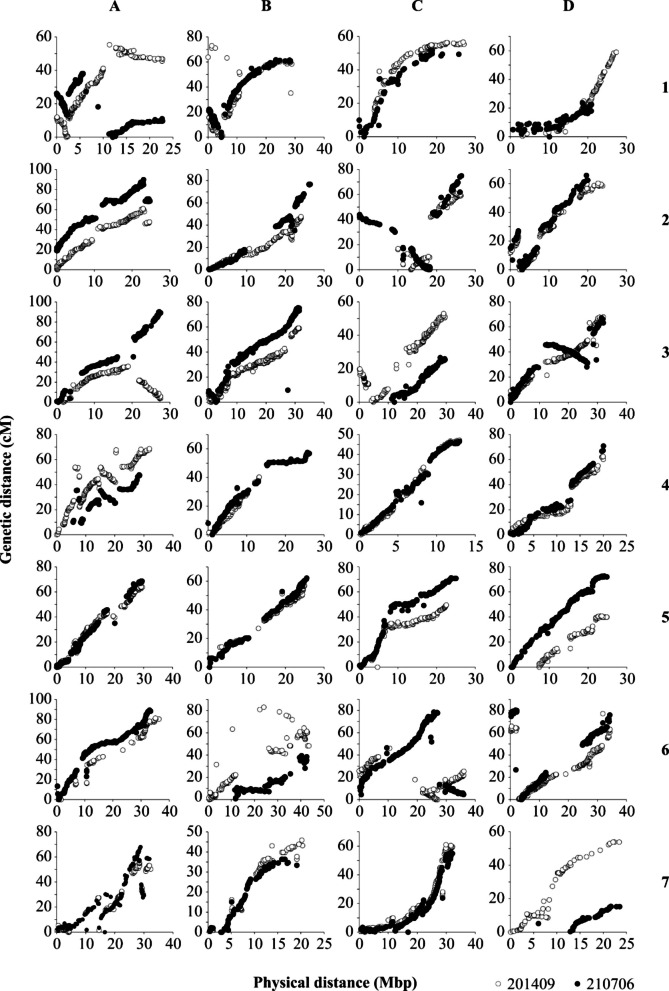
Comparison of genetic and physical positions of SNP markers, mapped on *F.* × *ananassa* 201409 (white) and 210706 (black) linkage groups. The physical position of the marker was determined by blasting marker sequence with the reference genome of *F.* × *ananassa* cv. ˈCamarosaˈ v.1.0 genome assembly [21]. Linkage groups of the homoeologous groups 1–7 were named according to the recent *F.* × *ananassa* nomenclature A-D [26]

### QTL analysis

Multiple QTLs were identified for WS and P_f_ traits in both years and on both parental maps (Tables 5 and 6). With the phenotypic data from 2024, mostly identical QTL regions were found on both parental maps on LG 1B (Fvb1-2), 3B (Fvb3-2), 4 C (Fvb4-2), and 5 A (Fvb5-1) for both traits (Table 5 and Fig. 3). Remarkably, all QTLs on 5A shared the SNP marker AX-184113813 in the same genetic and physical position on both maps. On LG 1B, two QTLs were detected on both parental maps (1Ba and 1Bb, Fig. 3) for both traits. With the maternal map, only *qP*_*f*_*−1Ba-2024* was found for P_f_; the second QTL region was not significant due to a LOD score below the LOD_CW_ threshold. The significance level of the detected marker-phenotype associations was mostly high for QTLs on LG 3B, 4C, and 5A (*p* < 0.01), but not for 1B, 1C, and 7C (*p* < 0.1). Most of the identified QTLs in season 2024 did not exceed the LOD_GW_ thresholds, except for QTL *qP*_*f*_*−1Ba-2024* for 201706, explaining 28.6% of the phenotypic variance (LOD_max_ = 5.18). The LOD_max_ scores of QTLs on 1B and 3B were appreciably higher than the corresponding LOD_CW_, accompanied by a high percentage of variance explanation thresholds (e.g., *qWS-1Ba-2024* of 201409, LOD_max_ = 4.61, %Exp. = 43.1 or *qPf-3B-2024* of 210706, LOD_max_ = 4.10, %Exp. = 23.4). Other QTLs on LG 4 C, 5 A, and 7 C only slightly exceeded the LOD_CW_ threshold and showed a lower percentage of variance explanation (e.g., *qWS-7C-2024* of 201409, LOD_max_ = 3.18, %Exp. = 18.4 or *qP*_*f*_*−4C-2024* of 210706, LOD_max_ = 3.13, %Exp. = 18.4).

**Table 5:** List of QTLs found on parental maps of *F.* × *ananassa* 210409 × 210706 F_1_ population for water soaking (WS) and fruit skin permeance in water uptake (P_f_) in season 2024 (n F_1_ = 71). Plants were grown in a greenhouse. Linkage groups of the homoeologous groups 1–7 were named according to the recent *F.* × *ananassa* nomenclature A-D [26]. For each SNP marker with the highest LOD score, chromosome name and physical position was given, based on the *F.* × *ananassa* ˈCamarosaˈ v.1.0 genome assembly [21]. Level of significance and K-value were determined using a Kruskal–Wallis test and genome-wide (GW) and chromosome-wide (CW) LOD thresholds were calculated using a permutation test. By interval mapping, the highest LOD score (LOD_max_) and percentage of variance explained (Exp.%) were estimated. The QTL region, including the markers genetic position, was calculated with LOD_max_−1

				***F*x*a* ˈCamarosaˈ**		**Kruskal–Wallis**		**Permutation test**		**Interval mapping**		**Genetic map**	
**Trait**	**Parent**	**QTL ID**	**Marker ID**	**Chr**	**Position (bp)**	**K**	**Signif**	**LOD**_**GW**_	**LOD**_**CW**_	**LOD**_**max**_	**Exp.%**	**Position (cM)**	**Region (cM)**
WS	201409	*qWS-1Ba-2024*	AX-184907012	Fvb1-2	7817305	4.75	*	5.1	3.5	4.61	43.1	22.025	18.241–22.025
		*qWS-1Bb-2024*	AX-184380336	Fvb1-2	10911051	5.82	**		3.5	4.24	24.1	50.381	50.381–51.910
		*qWS-3B-2024*	AX-184055455	Fvb3-2	6813220	13.92	*****		3.3	3.82	22	14.250	2.528–29.606
		*qWS-4C-2024*	AX-184740352	Fvb4-2	2371522	11.89	****		2.9	3.13	18.4	6.635	4.316–9.801
		*qWS-5A-2024*	AX-184113813	Fvb5-1	29120201	12.83	****		3.2	3.42	19.9	63.937	58.676–64.075
		*(qWS-7C-2024)*	AX-184061493^†^	Fvb7-1	31024238	3.35	*		3.1	3.18	18.4	56.135	54.32–60.997
	210706	*qWS-1Ba-2024*	AX-184537513	Fvb1-2	7477259	5.39	*	5.2	3.2	3.88	22.4	28.405	18.361–33.770
		*qWS-1Bb-2024*	AX-184915866	Fvb1-2	12257932	12.36	******		3.2	4.34	25.7	44.003	43.792–47.011
		*(qWS-1C-2024)*	AX-184279098	Fvb1-3	9872635	4.97	*		3.5	3.63	40.8	33.892	31.399–34.013
		*qWS-3B-2024*	AX-184055455	Fvb3-2	6813220	13.92	*****		3.6	4.15	23.7	22.495	9.577–28.519
		*qWS-4C-2024*	AX-184593687	Fvb4-2	2397714	11.61	****		3	3.13	18.4	7.566	4.657–11.734
		*qWS-5A-2024*	AX-184113813	Fvb5-1	29120201	12.83	****		3.2	3.25	19	68.330	63.479–68.725
P_f_	201409	*qP*_*f*_*−1Ba-2024*	AX-184065984^†^	Fvb1-2	7214467	6.32	**	5.1	3.6	4.21	24	18.323	14.567–22.045
		*qP*_*f*_*−3B-2024*	AX-184505625	Fvb3-2	4434681	12.75	****		3.3	4.17	23.7	4.195	2.125–19.774
		*qP*_*f*_*−4C-2024*	AX-184428375	Fvb4-2	2303388	10.27	***		3	3.13	18.4	5.755	3.115–12.158
		*qP*_*f*_*−5A-2024*	AX-184113813	Fvb5-1	29120201	12.68	****		3.2	3.25	19	63.937	59.663–64.075
	210706	***qP***_***f***_***−1Ba-2024***	**AX-184537513**	**Fvb1-2**	**7477259**	**11.40**	****	**5**	**3.2**	**5.18**	**28.6**	**28.405**	**27.082–28.489**
		*qP*_*f*_*−1Bb-2024*	AX-184545559	Fvb1-2	12402863	9.03	****			3.6	21.9	43.792	43.792–44.003
		*qP*_*f*_*−3B-2024*	AX-184177183	Fvb3-2	3858636	8.71	****		3.5	4.1	23.4	9.780	7.811–14.449
		*qP*_*f*_*−4C-2024*	AX-184428375	Fvb4-2	2303388	10.27	***		3	3.13	18.4	6.777	4.041–13.422
		*qP*_*f*_*−5A-2024*	AX-184113813	Fvb5-1	29120201	12.68	****		3.1	3.18	18.6	68.33	64.518–68.725

Bold: QTL exceeded LOD_GW_ threshold; QTLs in brackets were only found in one of the parents

^†^Peak marker was not significant

*p* < 0.1, *; *p* < 0.05, **; *p* < 0.01, ***; *p* < 0.005, ****; *p* < 0.001, *****; *p* < 0.0005, ******; *p* < 0.0001, *******

**Table 6:** List of QTLs found on parental maps of *F.* × *ananassa* 210409 × 210706 F_1_ population for water soaking (WS) and logarithmic transformed fruit skin permeance in water uptake (log P_f_) in season 2025 (n F_1_ = 128). Plants were grown in a high tunnel table-top system under open-field conditions. Linkage groups of the homoeologous groups 1–7 were named according to the recent *F.* × *ananassa* nomenclature A-D [26]. For each SNP marker with the highest LOD score, chromosome name and physical position was given, based on the *F.* × *ananassa* ˈCamarosaˈ v.1.0 genome assembly [21]. Level of significance and K-value were determined using a Kruskal–Wallis test and a genome-wide (GW) and chromosome-wide (CW) LOD thresholds were calculated using a permutation test. By interval mapping, the highest LOD score (LOD_max_) and percentage of variance explained (Exp.%) were estimated. The QTL region, including the markers genetic position, was calculated with LOD_max_−1

				***F*x*a* ˈCamarosaˈ**		**Kruskal–Wallis**		**Permutation test**		**Interval mapping**		**Genetic map**	
**Trait**	**Parent**	**QTL ID**	**Marker ID**	**Chr**	**Position (bp)**	**K**	**Signif**	**LOD**_**GW**_	**LOD**_**CW**_	**LOD**_**max**_	**Exp.%**	**Position (cM)**	**Region (cM)**
WS	210409	*qWS-1Bb-2025*	AX-184743927	Fvb1-2	10752123	3.75	*	4.8	3.4	3.44	11.7	51.910	49.202–51.910
		*qWS-2A-2025*	AX-184883855	Fvb2-2	4320490	14.97	*****		3	3.87	13	17.811	15.694–23.129
		*(qWS-3A-2025)*	AX-184059539	Fvb3-4	21846270	10.39	***		3.4	3.47	14.3	21.376	19.909–21.500
		*qWS-7B-2025*	AX-184074753^†^	Fvb7-3	16158679	13.86	*****		3.1	4.59	15.4	39.817	33.094–41.811
		*qWS-7C-2025*	AX-184367516^†^	Fvb7-1	29618275	4.29	**		3	3.9	13.2	56.916	51.919–60.997
	210706	*qWS-1Bb-2025*	AX-184894612	Fvb1-2	13824100	11.38	*****	4.8	3.1	3.28	13.2	47.480	46.226–54.970
		*qWS-2A-2025*	AX-184883855	Fvb2-2	4320490	14.97	*****		3.1	3.88	13	40.719	39.876–46.323
		*qWS-7B-2025*	AX-184147044^†^	Fvb7-3	17062838	16.78	*******		2.9	4.6	15.3	34.359	31.849–36.363
		*qWS-7C-2025*	AX-184699875	Fvb7-1	29761498	10.46	****		3	4.19	14.2	50.319	46.43–55.624
logP_f_	201409	*(qlogP*_*f*_*−1D-2025)*	AX-184300776	Fvb1-1	3477773	3.02	*	4.7	2.9	3.47	13.7	1.518	1.504–3.991
		***qlogP***_***f***_***−2A-2025***	**AX-184646139**	**Fvb2-2**	**6086274**	**20.21**	*******		**3**	**5.08**	**16.7**	**21.205**	**15.694–29.919**
		*qlogP*_*f*_*−7C-2025*	AX-184367516^†^	Fvb7-1	29618275	3.08	*		3.1	4.03	13.5	56.916	51.265–57.446
	210706	***qlogP***_***f***_***−2A-2025***	**AX-184646139**	**Fvb2-2**	**6086274**	**20.21**	*******	**4.7**	**3.2**	**4.97**	**16.4**	**44.639**	**39.876–52.086**
		*qlogP*_*f*_*−7C-2025*	AX-184699875	Fvb7-1	29761498	10.43	****		3.1	4.17	14	50.319	47.084–51.960

Bold: QTL exceeded LOD_GW_ threshold; QTLs in brackets were only found in one of the parental maps

^†^Peak marker was not significant

*p* < 0.1, *; *p* < 0.05, **; *p* < 0.01, ***; *p* < 0.005, ****; *p* < 0.001, *****; *p* < 0.0005, ******; *p* < 0.0001, *******

**Fig. 4:**
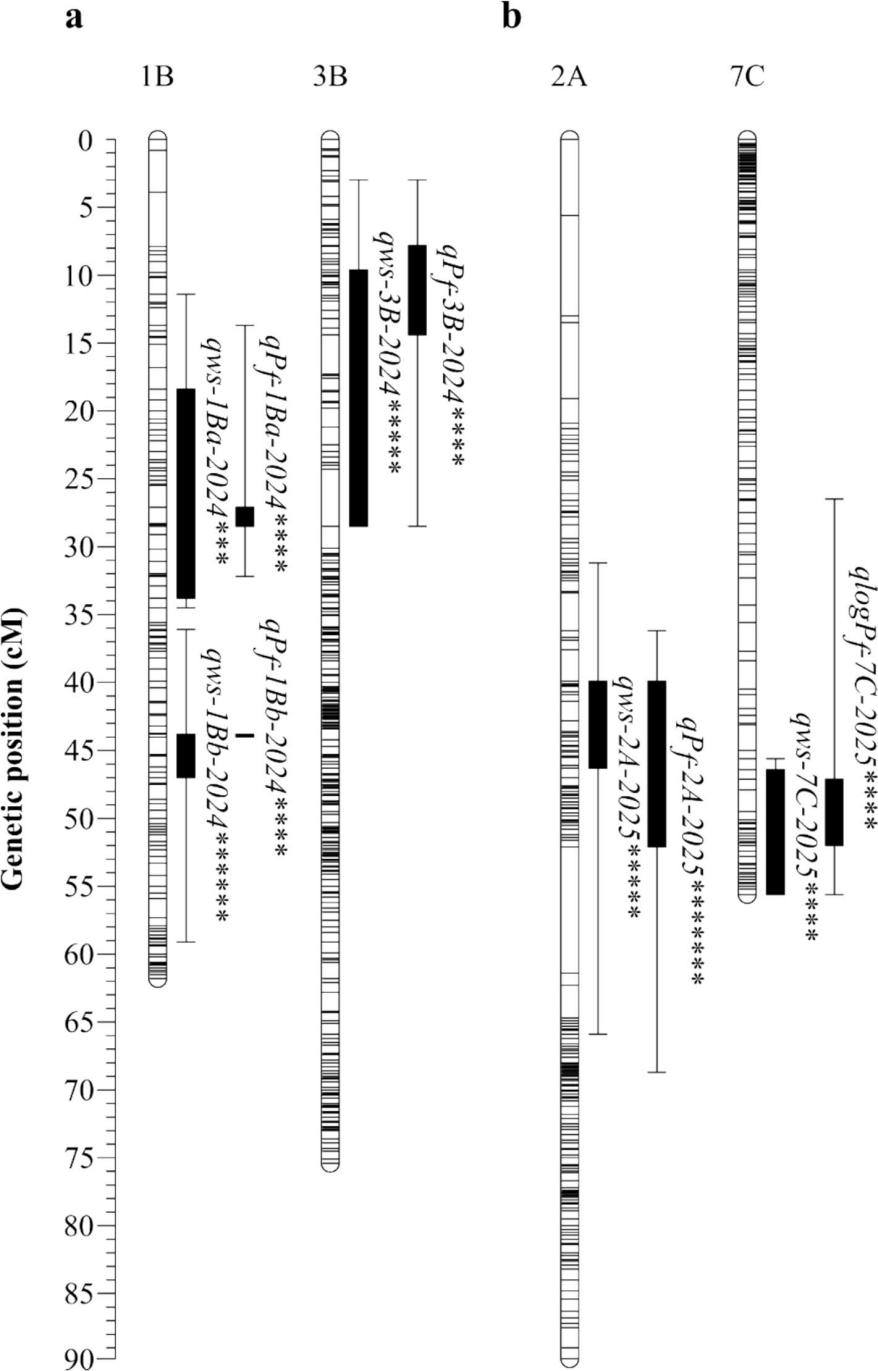
Selection of QTLs found on paternal map of *F.* × *ananassa* 210706 for water soaking (WS), fruit skin permeance (P_f_) and log transformed fruit skin permeance (log P_f_). Phenotyping in a *F*. × *ananassa* F_1_ 201409 × 210706 cross population was performed over two years (2024, *n* = 71, 2025, *n* = 128). WS was indexed after 4 h incubation in deionized water using a 5-point rating scale: score 0, no WS; score 1, < 10% of the fruit surface area water-soaked; score 2, 10–35%; score 3, 36–60%; score 4, > 60% [30] and P_f_ was calculated as described in Hurtado et al. [36]. **a** In 2024, plants were kept under greenhouse conditions. **b** In 2025, plants were grown in a high tunnel table-top system under open-field conditions. Linkage groups of the homoeologous groups 1–7 were named according to the recent *F.* × *ananassa* nomenclature A-D [26]. Level of significance was determined with a Kruskal–Wallis test (*p* < 0.01, ***; *p* < 0.005, ****; *p* < 0.001, *****; *p* < 0.0005, ******; *p* < 0.0001, *******). The QTL region with its inner and outer boundaries, was calculated with LOD_max_−1 and LOD_max_−2

With the phenotypic data of 2025 which included 128 individuals, the number of detected QTLs and their percentage of variation explanation was lower (%Exp. < 17%, Table 6). Consistent QTLs were only found in the second region of LG 1B for WS on both parental maps (*qWS-1Bb-2025*) and on LG 7C for both traits on both maps (e.g., *qWS-7C-2025*). A completely different QTL with a high marker-phenotype association (*p* < 0.001) was detected in the same region on maternal and paternal LG 2A for WS and P_f_, explaining 13–16.7% of phenotypic variation (Fig. 3). For WS, but not for P_f_, there was another QTL on LG 7B (*qWS-7B-2025*), explaining about 15.3% of the phenotypic variation. As the P_f_ values were not normally distributed in 2025 (skewness = 1.25; kurtosis = 2.07; Shapiro–Wilk W test statistic = 0.92, *p* < 0.001), a logarithmic transformation was done. Transformed P_f_ values led to the detection of the same QTL as without transformation (not shown), but the effect of the QTL *qlogP*_*f*_*-2A-2025* was stronger, and the LOD_GW_ was exceeded for both parental maps (LOD_GW_ = 4.7; LOD_max_ 201409 = 5.08; LOD_max_ 210706 = 4.97). The significance of the QTLs found on LG 7C was higher on the paternal map (*p* < 0.005, Fig. 3) than on the maternal map (*p* < 0.05) for WS and P_f_. Other marker associations on LG 2A or 7B were highly significant (*p* < 0.001). Furthermore, two small QTL regions were identified on the maternal LG 3A for WS (size = 1.6 cM, %Exp. = 14.3) and on LG 1D for log-transformed P_f_ (size = 2.5 cM, %Exp. = 13.7). Compared to season 2024, most of the QTLs clearly exceeded the LOD_CW_ thresholds, except for *qWS-1Bb-2025* on both maps. The QTLs on both parental LG 7B almost exceed the LOD_GW_ threshold (e.g., *qWS-7B-2025* for 201409 LOD_max_ = 4.6 vs. LOD_GW_ = 4.8).

The number of genes found within the identified QTL regions above LOD_CW_ threshold varied based on season and parental map (Table S2 and S3, Supplementary Information). The maximum number of genes was found on the reference genome of ˈCamarosaˈ for QTL *qP*_*f*_*-3B-2024* with 1099 and *qlogP*_*f*_*-2A-2025* with 998. Only one single gene was found in the QTL *qWS-1C-2024* (ˈCamarosaˈ FxaC_3g18940 and ˈRoyal Royceˈ Fxa1Cg101526; predicted molybdate-anion transporter) and *qWS-3A-2025* (ˈCamarosaˈ FxaC_9g39840 and ˈRoyal Royceˈ Fxa3Ag101412; predicted probable potassium transporter 13 isoform X1). The marker with the highest LOD score of the significant paternal QTL *qP*_*f*_*-1Ba-2024* aligned near the region of FxaC_2g16570 and Fxa1Bg200964 on both reference genomes, which encodes for an aquaporin PIP2-7-like protein. The QTL *qlogP*_*f*_*-2A-2025* peak position of AX-184646139, found on both parental maps, was located near ˈCamarosaˈ FxaC_5g13140 or ˈRoyal Royceˈ Fxa2Ag102324, which encodes a predicted serine/threonine-protein kinase HT1.

## Discussion

### A segregating F_1_ population formed the basis of linkage mapping and QTL analysis in this study

The progenies derived from the two contrasting parents varied in WS susceptibility. Both parental genotypes were previously reported in Hurtado et al. [36] as showing contrasting susceptibilities to WS. In our study, the WS values of both parents were similar to what was previously reported [36], and significant differences reflected their contrasting characters, which justify their selection as parental genotypes of a mapping population.

The application of molecular markers allowed for the identification of selfers and outcrossers within the progenies. Although the complexity of the allo-octoploid genome of *F.* × *ananassa* made the analysis challenging, the SSR marker data and the SNP data were in good agreement. Marker analysis of polyploid genomes is often impaired by allele dosage uncertainty, and homoplasy (presence of null alleles and replication slippages), which could lead to a loss of information due to inaccurate allele frequency calculation [20]. The *polysat* package [15, 16] allowed us to compute allele frequencies and phylogenetic population structure according to the ploidy level.

A surprisingly high proportion of genotypes with only maternal alleles was detected among the obtained F_1_ population. This subpopulation was referred to as selfers. It is conceivable that the protection against self-pollination failed due to late castration of the male anthers during the process of artificial fertilization of the maternal flower. After exclusion of non-biparental genotypes, a total of 136 F_1_ individuals remained for phenotyping for WS and P_f_ over two seasons. This population clearly segregated in both traits. The sample size of phenotyped F_1_ progenies was remarkably smaller in 2024 due to the exclusion of selfers, low number of plants per genotype and insufficient flower induction of the established biparental seedlings. The vegetative propagation by runners enabled sufficient fruit production in 2025 by multiple plants per genotype and enlarged the number of F_1_ progenies, which could be phenotyped successfully.

### Linkage map quality plays an important role in QTL analysis

The quality of the resulting parental maps was restrained by uneven marker distribution with large gaps, segregation distortion (SD), and sometimes poor collinearity of genetic and physical marker positions. Several factors affect the quality of mapping. Marker polymorphism is crucial for mapping [9]. Whereas the SNP markers of the categories PHR (co-dominant) and NMH (dominant) were known to be highly polymorphic [24], this must be assessed individually for each SSR marker. With the calculation of the H_exp_ and PIC values, it is possible to gain information about the polymorphism of the individual marker and its possible usage for linkage mapping [10, 43].

The quality of the genetic map also depends on the population size, influencing marker order and genetic distance calculation [17]. Thus, inaccurate mapping results could be related to the unexpectedly lower number of F_1_ genotypes due to the excluded selfing subpopulation.

The genetic and physical marker position is linked non-linearly [9] and also depends on genome size and presence of regions with high recombination frequency [17]. For many linkage groups, there was a good collinearity between the genetic and physical positions. However, in some linkage groups, rearrangements were observed (LG 1A, 1B, 2C, 2D, 6C, and 6D), corresponding to intra-chromosomal scaffolding errors in the reference genome ˈCamarosaˈ v.1.0 [24]. For other rearrangements in LG 3D, 4A, 6B, and 7A, errors in genotyping could be assumed. Additionally, SD could influence linkage mapping, and it could occur due to many intrinsic and extrinsic reasons [9].

### Similar genetic regions are involved in both WS susceptibility and fruit skin permeance P_f_

Independent of the genetic background, WS and P_f_ were closely related, and the underlying mechanisms were assumed to be the same [36]. This could be confirmed in this study by the significant coefficient of correlation, which was of a similar margin as in Hurtado et al. [36]. Both WS and P_f_ traits differ in their type of measurement. WS is an ordinal trait determined by rating the severity of symptoms [30]. In contrast, fruit skin permeance P_f_ is a continuous variable calculated based on flow rates, fruit mass and soluble solids [36]. Therefore, it was also of interest to explore if similar genetic regions are also involved in accordance with the method. Indeed, the consistent association of both traits observed across seasons suggests that either a common gene exerts pleiotropic effects on both traits or that different genes are closely clustered within a narrow chromosomal region. The consistency of trait-related QTLs supported the relationship between WS and P_f_.

Many genes are involved in fruit cracking of several fruit crops, which was reviewed in [65]. But for cultivated strawberries, less information is available. In 2024, Straube et al. investigated the gene expression of cuticle-associated genes during fruit development of strawberries, and findings indicated a downregulation of several of those genes during full ripening [70]. Most of the genes involved in the study were not located on linkage groups where QTLs have been found. Nevertheless, two genes, *FaSHN2* (accession maker-Fvb2-2-augustus-gene-27.57) and *FaGPTA3* (accession maker-Fvb2-2-augustus-gene-102.37), were located on LG 2A (Fig. 4) [70], on which a significant QTL was found (*qlogP*_*f*_*-2A-2025*). Unfortunately, both genes were located inside map regions, where marker coverage was low. It could be worthwhile to design markers, which increase the resolution of the map areas and repeat QTL analysis. Furthermore, the physical position of the significant marker AX-184646139 of the QTL *qlogP*_*f*_*-2A-2025* was linked to a region where a serine/threonine-protein kinase HT1 is probably encoded [42], which is involved in plant defence and signalling [2], but not in cuticle-related processes.

**Fig. 5:**
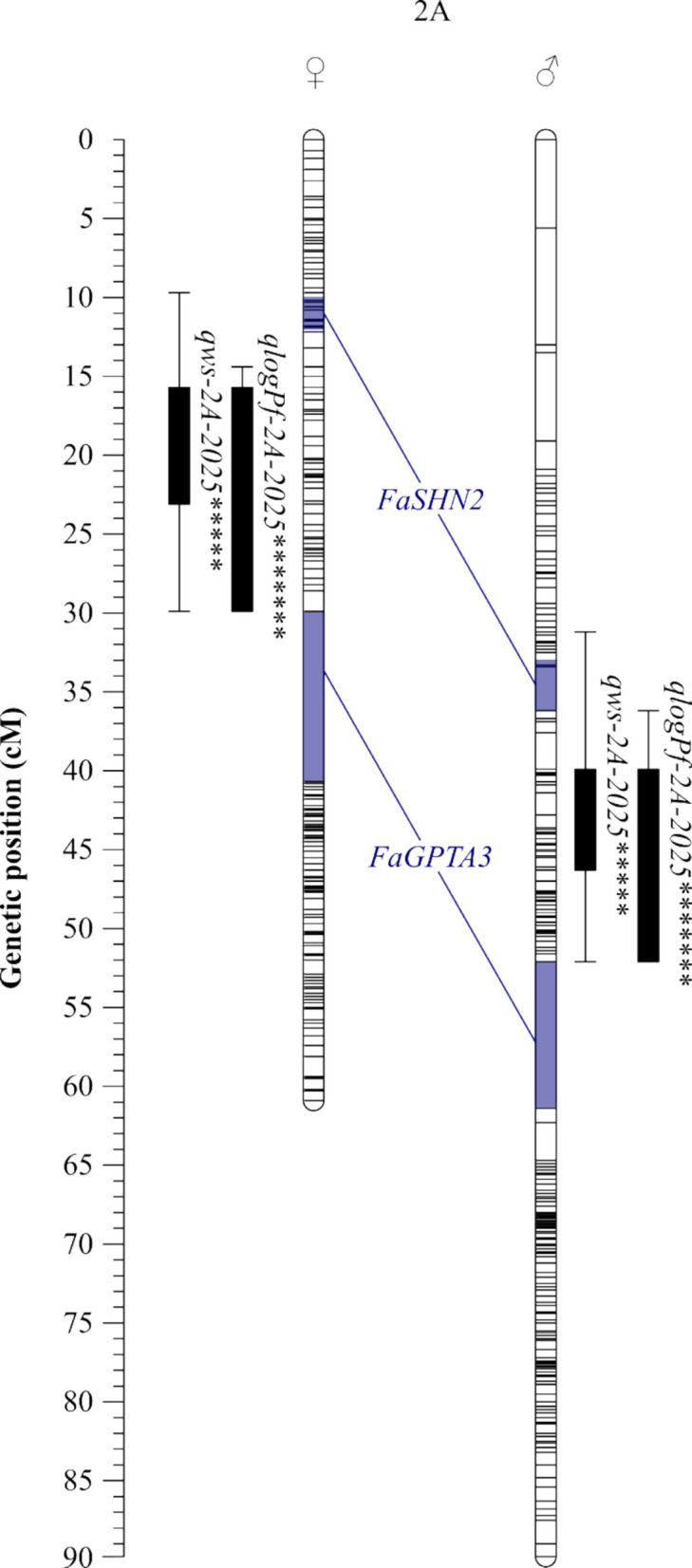
QTLs found on linkage group 2A of both parental maps of *F.* × *ananassa* for water soaking (WS) and logarithmic transformed fruit skin permeance (log P_f_). WS was indexed after 4 h incubation in deionized water using a 5-point rating scale: score 0, no WS; score 1, < 10% of the fruit surface area water-soaked; score 2, 10–35%; score 3, 36–60%; score 4, > 60% [30] and P_f_ was calculated as described in Hurtado et al. [36]. In 2025, plants were grown in a high tunnel table-top system under open-field conditions. Level of significance was determined with a Kruskal–Wallis test (*p* < 0.001, *****; *p* < 0.0001, *******). Blue: Regions were two cuticle-related genes *FaSHN2* and *FaGPTA3* could be located

The significant marker AX-184537513 of the paternal QTL *qP*_*f*_*-1Ba-2024* for P_f_ on LG 1B aligned near an aquaporin PIP2-7-like protein [42]. Plasma membrane intrinsic proteins (PIP) are membrane channels which facilitate water and nutrient transport [52]⁠. They were suggested to play a role in fruit cracking in jujube, litchi, apple, and sweet cherry [65]. In *F.* × *ananassa*, PIP1 and PIP2 co-expression resulted in enhanced plasma membrane permeability [4, 80], and it has been observed that a higher expression was coupled with a higher fruit firmness [4]. However, according to Chen et al. [14], water uptake through the fruit skin into the cells is a composite process comprising penetration through the cuticle, the cell wall and the plasma membrane. Of these ‘resistors’, the cuticle is by far the largest resistor and hence, penetration of the cuticle is the limiting step in water uptake. The permeances of the plasma membrane and cell wall are higher by orders of magnitude [66]. This makes an involvement of aquaporins in the cracking process unlikely [14]. Nevertheless, the QTLs found on LG 1B could represent two separate genomic regions, even if they have not always been detected because of a possible underestimation of linkage [45]. The large confidence intervals of the detected genomic regions are another common issue of QTL analysis with segregating populations [45], and so aquaporins and serine/threonine-protein kinases might play a role in the phenotypic variation of WS in strawberry, but both QTLs on LG 1B and 2A contain many more genes, and further studies are essential to select candidate genes.

### Water soaking and fruit skin permeance underlying polygenic and environmental effects

In nature, many traits have a polygenic background and the phenotypic variation is determined by multiple genetic loci with small effects [71].

The presence of multiple QTLs for WS and P_f_ on different linkage groups indicates that these traits are polygenic. Although gene × gene interactions are relevant, gene × environment interactions were more important for WS and P_f_-associated QTLs. This conclusion is based on the finding that different QTLs were detected in the two seasons. However, several factors must be considered. First, the number of genotypes had almost doubled in 2025 compared to 2024. Second, growth rates and the rates of surface expansion affect the rates of strain of the cuticle. Growth rates markedly depend on environmental factors such as temperature and water vapor pressure deficit. Third, microcracking of the cuticle, the first step in the development of WS, is affected by surface wetness duration. Surface wetness duration, in turn, may result from dew formation and high humidity, which are likely to vary with environmental conditions even in protected environments. These arguments indicate that the low reproducibility of the QTLs between the two seasons is not really surprising. Herrington et al. [28] were the first to calculate broad-sense heritability for rain damage in cultivated strawberry. Their estimates were similar to or slightly higher than those obtained in our study. These findings indicate the presence of gene × environment interactions that resulted in the lack of reproducible QTLs between the two seasons. However, QTL estimation is affected by multiple factors, including genetic map quality, environmental parameters, population size and errors in geno- and phenotyping [17, 45, 71, 79]. The low sample size in 2024 could lead to overestimation of the effect size of the QTLs [8, 78]. In fact, the proportion of phenotypic variation (Exp.%) was higher for QTLs found in season 2024 than in season 2025, and it could be assumed that for e.g., *qWS-1Ba-2024*, the phenotypic variation of Exp.% = 43.1 was overestimated. Furthermore, the detected QTLs with minor effect and LOD scores slightly above LOD_CW_ thresholds could potentially be false positive QTLs (Type I error) [41].

## Limitations of the study and conclusions

Although our study provides first insights into the genetic basis of water soaking in strawberry, several factors limit this study and therefore highlight key areas for further research. Firstly, the population structure with the high proportion of selfers revealed possible inaccuracies during the crossing procedure (e.g., insufficient or delayed emasculation), which has to be improved for further crossing experiments. Secondly, there were differences in growth conditions and phenotyping procedures between both seasons of phenotyping as well as a small sample size in the first year of phenotyping, which has an effect on the phenotypic variation of the WS susceptibility and P_f_ and could influence the reproducibility of QTL detection. Therefore, further work will focus on phenotypic repetitions over several years with the same growth conditions, to allow for a full understanding of the genetic basis of this complex trait. Thirdly, genotyping with the Axiom™ Strawberry FanaSNP 50K array, unfortunately, did not result in the desired marker density, which impaired the map resolution. This resulted in large QTL intervals, making it impossible to precisely determine QTL regions and accurately determine candidate genes. Further study will also focus on fine-mapping QTL regions by significantly increasing the mapping population, as well as the development of markers in and around QTL intervals.

In conclusion, water soaking is an important disorder in the open-field production of strawberries, and breeding of tolerant cultivars is crucial for reducing pre- and postharvest fruit losses. Our findings indicate that susceptibility to WS is a complex trait that is polygenically controlled and strongly influenced by gene × environment interactions. To our knowledge, this is the first study of the genetic basis of WS in strawberries. The detected QTLs could be indicative of the genetics underlying these traits. However, follow-up studies are necessary. An additional phenotyping with a higher number of phenotyped genotypes will help to reduce the bias and false positive QTLs.

Finally, a validation of the QTLs in an independent cross-population is needed, as well as a better resolution of the QTL regions by developing additional markers. Furthermore, multi-environmental and multi-seasonal validations will help to confirm the robustness of the QTLs. This study implements initial key steps towards marker-assisted selection of tolerant cultivars.

## Supplementary Information

Supplementary Material 1: Figure S1: Population structure of *F*. × *ananassa* F_1_ 201409 × 210706 cross population. Table S1: List of SSR primer pairs for multiplex PCR (MP-PCR) and fragment analysis. Table S2: List of QTLs and their underlying genes found on parental maps of *F*. × *ananassa* F_1_ 201409 × 210706 cross population for water soaking (WS) and fruit skin permeance (P_f_) in season 2024. Table S3: List of QTLs and their underlying genes found on parental maps of *F*. × *ananassa* F_1_ 201409 × 210706 cross population for water soaking (WS) and logarithmic transformed fruit skin permeance (log P_f_) in season 2025.

Supplementary Material 2: Table S4: Original Genotyping data from the Axiom™ Strawberry FanaSNP 50K Genotyping Array, Thermo Fisher Scientific Inc. [24]. Table S5: Phenotypic data of F1 progenies of a *F*. × *ananassa* 201409 × 210706 cross population used for QTL analysis.

## Data Availability

The datasets generated in this study are published herein and also available as supplementary information. The original SNP data and the phenotypic data are presented as supplementary materials.
