# Supplementary material for: Water soaking in strawberry (Fragaria × ananassa) has a polygenic background and is strongly influenced by environmental factors
Source: BMC Plant Biol. 2026 May 11;26:828. doi: 10.1186/s12870-026-08778-2 (PMC13159323; doi:10.1186/s12870-026-08778-2)
Supplement: Supplementary file 1 — Supplementary Material 1: Figure S1: Population structure of F. × ananassa F1 201409 × 210706 cross population. Table S1: List of SSR primer pairs for multiplex PCR (MP-PCR) and fragment analysis. Table S2: List of QTLs and their underlying genes found on parental maps of F. × ananassa F1 201409 × 210706 cross population for water soaking (WS) and fruit skin permeance (Pf) in season 2024. Table S3: List of QTLs and their underlying genes found on parental maps of F. × ananassa F1 201409 × 210706 cross population for water soaking (WS) and logarithmic transformed fruit skin permeance (log Pf) in season 2025. [file 12870_2026_8778_MOESM1_ESM.docx]

**Water soaking in strawberry (*Fragaria × ananassa*) has a polygenic background and is strongly influenced by environmental factors**

Molecular breeding

Diana Seidler^1, 2^, Moritz Knoche^1^, Klaus Olbricht^3^, Henryk Flachowsky^2^, Ofere Francis Emeriewen^2^

^1^Gottfried Wilhelm Leibniz University Hannover, Institute for Horticultural Production Systems, Hannover, Germany

^2^Julius Kühn Institute, Institute for Breeding Research on Fruit Crops, Dresden, Germany

^3^Hansabred GmbH & Co. KG, Dresden, Germany

Email addresses of corresponding authors: diana.seidler@obst.uni-hannover.de; ofere.emeriewen@julius-kuehn.de


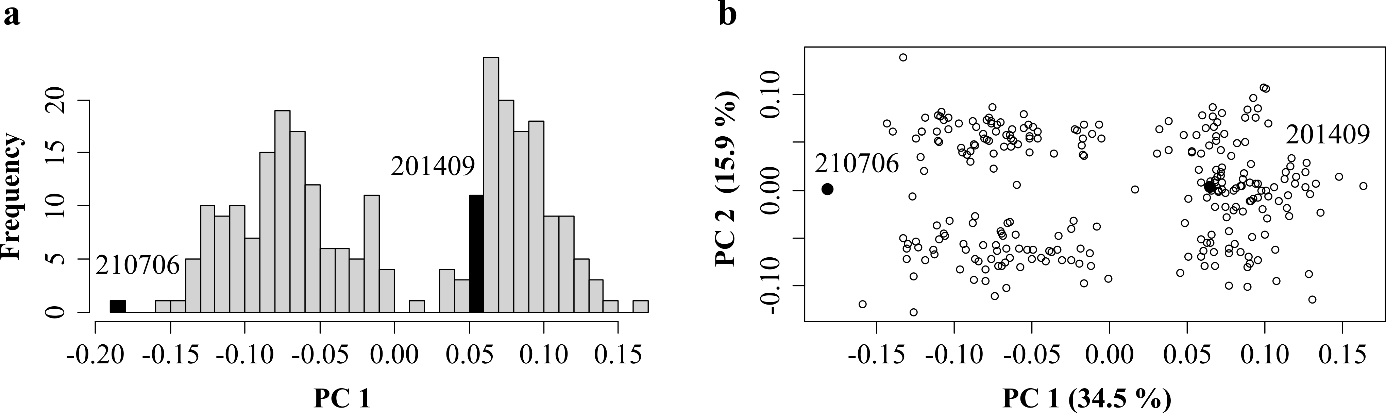


**Fig. S1** Population structure of *F*. × *ananassa* F_1_ 201409 × 210706 cross population. (a) Distribution of pairwise genetic distances between individuals. Black bars contain parental genotypes 201409 (maternal) and 210706 (paternal). (b) Principal coordinate analysis with the first two principal coordinates (PC, in brackets: proportion of variation explained by PC 1 and PC 2). A pairwise Bruvo distance matrix, calculated with the R *polysat* package (Clark and Jasieniuk 2011; Clark and Schreier 2017) formed the basis. Black dots represented the parental genotypes 201409 and 210706


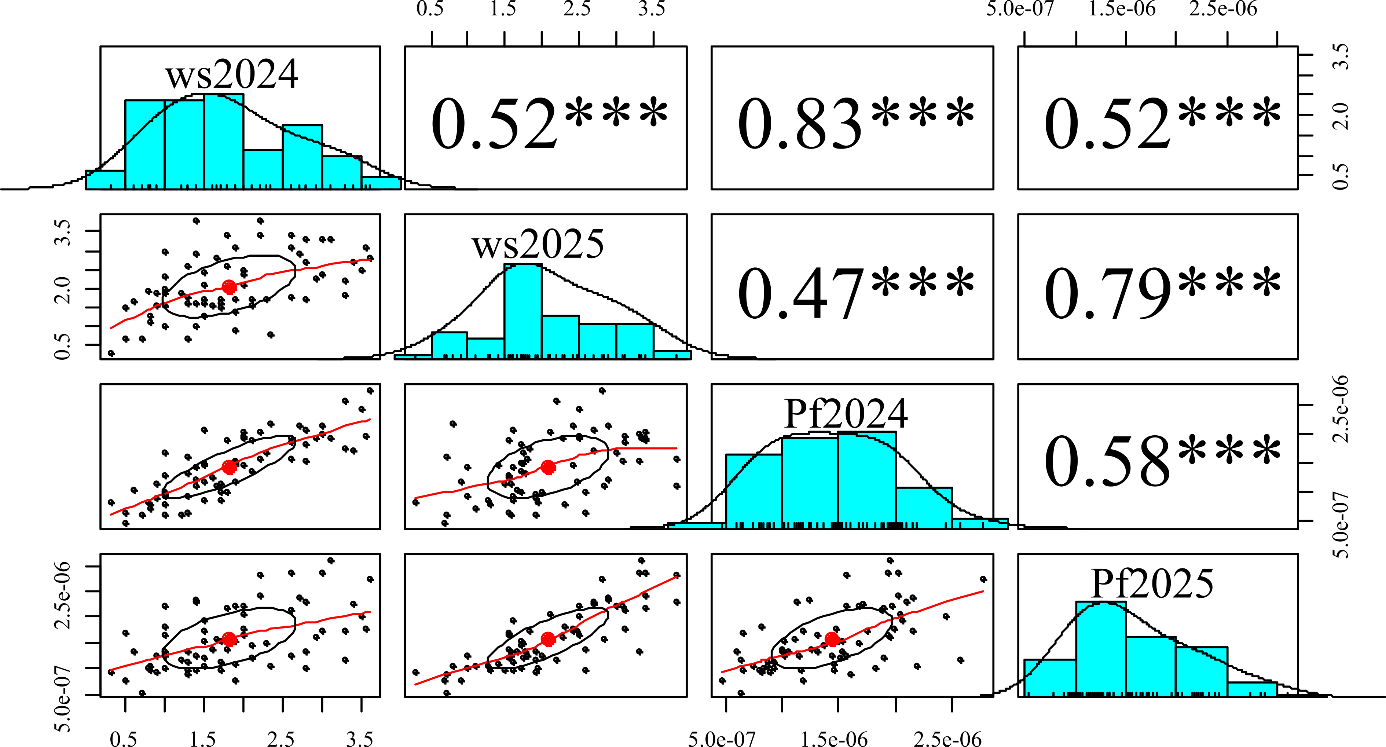


**Fig. S2** Correlation matrix after Spearman between water soaking susceptibility (WS, score) and fruit skin permeance for water uptake (P_f_, 10^-6^ m s^-1^). In 2024 and 2025, genotypes of *F*. × *ananassa* F_1_ 201409 × 210706 cross population were phenotyped for WS and P_f_. WS was indexed after 4 h incubation in deionized water using a 5-point rating scale: score 0, no WS; score 1, < 10% of the fruit surface area water-soaked; score 2, 10 - 35%; score 3, 36 - 60%; score 4, > 60% (Hurtado and Knoche 2021) and P_f_ was calculated as described in Hurtado et al. (2024). The diagonal showed the distribution of each trait per season. In the top-right part, the correlation coefficients after Spearman were shown with significance level (***, p < 0.001). The bottom-left part showed the bivariate scatterplots with a trend line. The means of 69 genotypes were used for correlation analysis with R package *psych* (Revelle W. 2026).

**Table S1** List of SSR primer pairs for multiplex PCR (MP-PCR) and fragment analysis (GenBank accession number, primer sequences 5’-3’, repeated motif, and references. Each forward primer was modified with a fluorescent dye label on the 5’-end (FAM, blue; ATTO532, green; ATTO550, yellow und ATTO565, red). One MP-PCR included four primer pairs. Reverse primers were pig-tailed (5’-GTTTCTT-3’)

| **Marker** | **Accession** | **Primer sequences forward** | **Primer sequences reverse** | **Motif** | **References** |
| --- | --- | --- | --- | --- | --- |
| UFFa_13C07_FAM | CO817563 | GGAGTCAACAGTAGTGCAGGTAA | GGTTTTCTTGCAGTTGGAGTAG | (CAG)_6_ | Bassil et al. (2006a) |
| CFVCT028_ATTO532 | DQ117035 | GGGAAGAGAGGCCTAAAACC | CGGCGTCTCAACTTGACC | (CT)_17_ | Monfort et al. (2006) |
| UDF-001_ATTO550 | BV097098 | ATCGAAGCACATGAAGAACG | TGGGTGGTGACTGGTGAGTA | (TG)_9_ | Cipriani and Testolin (2004) |
| UFFa_11G07_ATTO565 | CO817443 | TCTCTGTGTCTTCTCCGAAACT | CTACTGCTCCAACTTCAAATCG | (AT)_8_ | Bassil et al. (2006a) |
| CFVCT016_FAM | DQ117023 | CACAACGGAGTACACGGCTA | TCAGATTCACCTCGGACCTC | (GA)_18_ | Monfort et al. (2006) |
| UAFv7344_ATTO532 | D13991 | TCCTTTGTTTATTTGTATTGTTT | ATGATTGAAGTGGTGAAGATG | (TTCT)_4_ | Bassil et al. (2006b) |
| SF-A01_ATTO550 | GO479250 | GGGCAGCAACAAACCAAG | TAGGATGAACCACACTCTGAA | (TAC)_5_ | Njuguna (2010) |
| UFFa_09E12_ATTO565 | AJ870449 | CGAGGAAGTAACCTCACAGAAA | GGTGATGGAGAGTGCTGTTAGA | (AC)_6_ | Bassil et al. (2006a) |
| EMFv030_FAM | AJ564188 | TTCCGGAACAACAGCAACAAAG | GCGGCGGCACCATCTCG | (GGC)_5_ | Hadonou et al. (2004) |
| ChFaM036_ATTO532 | GU815797 | GCAGCCTCAAGAAGTGAAGG | CCATCTTGATATCACAGGCATA | (ATG)_9_ | Zorrilla-Fontanesi et al. (2011) |
| UDF-009_ATTO550 | BV097106 | CCTAGAGGAAAACACTGATGACTGA | AAGGCGAATGCTTTGGTATG | (AC)_17_ | Cipriani and Testolin (2004) |
| UDF-004_ATTO565 | BV097101 | GCTTGCATTTCAATAGCTGGA | TTTACTGATGCAGGAGTAGAATGA | (GT)_11_ | Cipriani and Testolin (2004) |
| EMFv017_FAM | AJ564176 | CCGACGAAACCAAGCACCTCCTAC | TGCATGATCAGCTACGACCTCCTC | (CCG)_6_ | Hadonou et al. (2004) |
| UFFa_01D03_ATTO532 | CO816689 | TTACTGAAATGGGTTTCAGAGC | GACAGCACAGTCATGGAAGATG | (TCT)_5_ | Bassil et al. (2006a) |
| UAFv8316_ATTO550 | NM_115720 | CGGTTAAACCAGATTACAACTCTC | GATCGAGCCCTACCAATTCA | (TC)_8_ | Bassil et al. (2006b) |
| MCAD_FAC_001_ATTO565 | - | CCCTCTTCCCGTAAAGTCCA | CGGACTCACCGGTCACTAGA | - | unpublished, IFAPA, Spain |
| ChFaM138_FAM | GU815857 | GGCAGTAACCTCCAGAACTCTAA | TCGCATTTCCAGCCTTATCT | (CCG)_6_ | Zorrilla-Fontanesi et al. (2011) |
| EMFv1_ATTO532 | AJ508244 | GGAGTTCCGCCTGCATCTTCTTCA | CGCGGTCGAGGTGCTAATGCTGTA | (CGG)_7_ | James et al. (2003) |
| UFFa_03C04_ATTO550 | AJ870444 | CGGTTCAGCAGGAGAATAAAAC | GCCCCATACTACCATTATGACC | (GGA)_5_ | Bassil et al. (2006a) |
| CHFaM078_ATTO565 | GU815822 | CAGCCTCATTGCAAATCTGA | CTTACCGGTTTCGATGTGGT | (CCT)_5_ | Zorrilla-Fontanesi et al. (2011) |

**Table S2** List of QTLs and their underlying genes found on parental maps of *F.* × *ananassa* 210409 × 210706 F_1_ population for water soaking (WS) and fruit skin permeance (P_f_) in season 2024 (n F_1_ = 71). Plants were grown in a greenhouse. WS was indexed after 4 h incubation in deionized water using a 5-point rating scale: score 0, no WS; score 1, < 10% of the fruit surface area water-soaked; score 2, 10 - 35%; score 3, 36 - 60%; score 4, > 60% (Hurtado and Knoche 2021) and P_f_ was calculated as described in Hurtado et al. (2024). For each QTL, the region of the QTL (cM) above the chromosome-wide LOD threshold (LOD_CW_) and the physical positions (bp) on the belonging reference genome of *F*. × *ananassa* ˈCamarosaˈ Genome Assembly v1.0.a2 (Edger et al. 2019; Liu et al. 2021) or ˈRoyal Royceˈ (Hardigan et al. 2021) was given. The number of genes was determined with the JBrowse tool of the GDR database (Jung et al. 2019)

|  |  |  | **LOD>LOD_CW_** |  | ***F*x*a* ˈCamarosaˈ** |  |  | ***F*x*a ˈ*Royal Royceˈ** |  |
| --- | --- | --- | --- | --- | --- | --- | --- | --- | --- |
| **Trait** | **Parent** | **QTL** | **Region (cM)** |  | **Region (bp)** | **n Genes** |  | **Region (bp)** | **n Genes** |
| WS | 201409 | *qWS-1Ba-2024* | 18.241-22.045 |  | 7114181-7817305 | 129 |  | 5030144-5628126 | 110 |
|  |  | *qWS-1Bb-2024* | 50.381-51.91 |  | 10752123-10911051 | 30 |  | 8431609-8573053 | 20 |
|  |  | *qWS-3B-2024* | 14.25-19.811 |  | 6370369-7124636 | 115 |  | 6069034-6824746 | 107 |
|  |  | *qWS-4C-2024* | 4.661-9.801 |  | 1795014-3216022 | 263 |  | 23287351-24874959 | 263 |
|  |  | *qWS-5A-2024* | 63.054-63.937 |  | 28331448-29548160 | 187 |  | 25081041-25883567 | 97 |
|  |  | *qWS-7C-2024* | 56.054-56.135 |  | 31024238-31203120 | 30 |  | 21696353-21872818 | 29 |
|  | 210706 | *qWS-1Ba-2024* | 25.436-33.77 |  | 5492677-8696172 | 550 |  | 5040342-6538434 | 254 |
|  |  | *qWS-1Bb-2024* | 43.792-47.011 |  | 12257932-13763752 | 182 |  | 9732831-11181597 | 178 |
|  |  | *qWS-1C-2024* | 33.892 |  | 9872635 | 1 |  | 9437506 | 1 |
|  |  | *qWS-3B-2024* | 21.172-28.519 |  | 6813220-6971660 | 22 |  | 6508641-6665837 | 21 |
|  |  | *qWS-4C-2024* | 5.649-10.834 |  | 1795014-3216022 | 263 |  | 23287351-24874959 | 231 |
|  |  | *qWS-5A-2024* | 67.672-68.629 |  | 28331448-29094323 | 112 |  | 25081041-25858528 | 95 |
| P_f_ | 210409 | *qPf-1Ba-2024* | 17.435-18.936 |  | 6990757-7364641 | 78 |  | 4836731-5343867 | 96 |
|  |  | *qPf-3B-2024* | 3.27-11.735 |  | 316935-6578824 | 1099 |  | 3700708-6277178 | 417 |
|  |  | *qPf-4C-2024* | 5.08-7.366 |  | 1542083-2552286 | 189 |  | 24105530-25140203 | 176 |
|  |  | *qPf-5A-2024* | 63.709-63.9 |  | 29094323-29548160 | 76 |  | 25858528-26275150 | 70 |
|  | 210706 | ***qPf-1Ba-2024*** | **27.082-28.489** |  | **7438804-7552525** | **22** |  | **5297338-5335194** | **8** |
|  |  | *qPf-1Bb-2024* | 43.792-44.003 |  | 12257932-12402863 | 23 |  | 9732831-9863987 | 24 |
|  |  | *qPf-3B-2024* | 9.78-14.449 |  | 3858636-5211082 | 223 |  | 3600107-4962156 | 232 |
|  |  | *qPf-4C-2024* | 5.649-7.566 |  | 1795014-2397714 | 119 |  | 24874959-28105079 | 312 |
|  |  | *qPf-5A-2024* | 67.672-68.629 |  | 28331448-29094323 | 112 |  | 25081041-25858528 | 95 |
| Note: Bold labelled QTL exceeded genome-wide LOD_GW_ threshold. | | | | | | | | | |

**Table S3** List of QTLs and their underlying genes found on parental maps of *F.* × *ananassa* 210409 × 210706 F_1_ cross population for water soaking (WS) and logarithmic transformed fruit skin permeance (log P_f_) in season 2025 (n F_1_ = 128). Plants were grown in a high tunnel table-top system under open-field conditions. WS was indexed after 4 h incubation in deionized water using a 5-point rating scale: score 0, no WS; score 1, < 10% of the fruit surface area water-soaked; score 2, 10 - 35%; score 3, 36 - 60%; score 4, > 60% (Hurtado and Knoche 2021) and P_f_ was calculated as described in Hurtado et al. (2024). For each QTL, the region of the QTL (cM) above the chromosome-wide LOD threshold (LOD_CW_) and the physical position (bp) on the belonging reference genome of *F.* × *ananassa* ˈCamarosaˈ Genome Assembly v1.0.a2 (Edger et al. 2019; Liu et al. 2021) or ˈRoyal Royceˈ (Hardigan et al. 2021) was given. The number of genes was determined with the JBrowse tool of the GDR database (Jung et al. 2019)

|  |  |  |  |  |  |  |  |  |  |
| --- | --- | --- | --- | --- | --- | --- | --- | --- | --- |
|  |  |  | **LOD>LOD_CW_** |  | ***F*x*a* ˈCamarosaˈ** |  |  | ***F*x*a ˈ*Royal Royceˈ** |  |
| **Trait** | **Parent** | **QTL** | **Range (cM)** |  | **Range (bp)** | **n Genes** |  | **Range (bp)** | **n Genes** |
| WS | 210409 | *qWS-1Bb-2025* | 50.381-51.91 |  | 10752123-10911051 | 30 |  | 8573053-8963248 | 60 |
|  |  | *qWS-2A-2025* | 15.694-21.359 |  | 3753168-6429732 | 484 |  | 17334582-20117937 | 462 |
|  |  | *qWS-3A-2025* | 21.376 |  | 21846270 | 1 |  | 8996251 | 1 |
|  |  | *qWS-7B-2025* | 36.371-41.811 |  | 13211299-17872908 | 546 |  | 7050597-11593295 | 542 |
|  |  | *qWS-7C-2025* | 51.919-59.286 |  | 29370973-32086965 | 527 |  | 21615921-22753092 | 222 |
|  | 210706 | *qWS-1Bb-2025* | 46.226-47.48 |  | 13426571-14077036 | 74 |  | 10860390-11438938 | 68 |
|  |  | *qWS-2A-2025* | 40.716-44.741 |  | 4153830-6128569 | 373 |  | 17650374-19673399 | 337 |
|  |  | *qWS-7B-2025* | 31.849-36.363 |  | 12887990-17247582 | 512 |  | 7631056-11906097 | 507 |
|  |  | *qWS-7C-2025* | 46.43-55.624 |  | 29683774-32129880 | 481 |  | 21901861-22796150 | 179 |
| P_f_ | 201409 | *qlogP_f_-1D-2025* | 1.504-3.782 |  | 3317277-4292430 | 64 |  | 22442917-22888575 | 28 |
|  |  | ***qlogP_f_-2A-2025*** | **15.694-23.129** |  | **3753168-9440297** | **961** |  | **16900902-20117937** | **539** |
|  |  | *qlogP_f_-7C-2025* | 51.265-57.446 |  | 29370973-31424880 | 392 |  | 21615921-22493093 | 172 |
|  | 210706 | ***qlogPf-2A-2025*** | **40.17-52.086** |  | **3792058-9669171** | **998** |  | **14202706-20092043** | **962** |
|  |  | *qlogPf-7C-2025* | 49.525-51.96 |  | 29683774-31486463 | 353 |  | 21901861-22146637 | 43 |
| Note: Bold labelled QTL exceeded genome-wide LOD_GW_ threshold. | | | | | | | | | |

References

Bassil, N.V.; Gunn, M.; Folta, K. M.; Lewers, K. S. (2006a): Microsatellite markers for Fragaria from ‘Strawberry Festival’ expressed sequence tags. In *Molecular Ecology Notes* 6 (2), pp. 473–476. DOI: 10.1111/j.1471-8286.2006.01278.x.

Bassil, N.V.; Njuguna, W.; Slovin, J. P. (2006b): EST‐SSR markers from Fragaria vesca L. cv. Yellow Wonder. In *Molecular Ecology Notes* 6 (3), pp. 806–809. DOI: 10.1111/j.1471-8286.2006.01351.x.

Cipriani, G.; Testolin, R. (2004): Isolation and characterization of microsatellite loci in Fragaria. In *Molecular Ecology Notes* 4 (3), pp. 366–368. DOI: 10.1111/j.1471-8286.2004.00655.x.

Clark, Lindsay V.; Jasieniuk, Marie (2011): POLYSAT: an R package for polyploid microsatellite analysis. In *Molecular ecology resources* 11 (3), pp. 562–566. DOI: 10.1111/j.1755-0998.2011.02985.x.

Clark, Lindsay V.; Schreier, Andrea Drauch (2017): Resolving microsatellite genotype ambiguity in populations of allopolyploid and diploidized autopolyploid organisms using negative correlations between allelic variables. In *Molecular ecology resources* 17 (5), pp. 1090–1103. DOI: 10.1111/1755-0998.12639.

Edger, Patrick P.; Poorten, Thomas J.; VanBuren, Robert; Hardigan, Michael A.; Colle, Marivi; McKain, Michael R. et al. (2019): Origin and evolution of the octoploid strawberry genome. In *Nature genetics* 51 (3), pp. 541–547. DOI: 10.1038/s41588-019-0356-4.

Hadonou, A. M.; Sargent, D. J.; Wilson, F.; James, C. M.; Simpson, D. W. (2004): Development of microsatellite markers in Fragaria, their use in genetic diversity analysis, and their potential for genetic linkage mapping. In *Genome* 47 (3), pp. 429–438. DOI: 10.1139/G03-142.

Hardigan, Michael A.; Feldmann, Mitchell J.; Pincot, Dominique D.A.; Famula, Randi A.; Vachev, Michaela V.; Madera, Mary A. et al. (2021): Blueprint for Phasing and Assembling the Genomes of Heterozygous Polyploids: Application to the Octoploid Genome of Strawberry: bioRxiv.

Hurtado, Grecia; Knoche, Moritz (2021): Water Soaking Disorder in Strawberries: Triggers, Factors, and Mechanisms. In *Frontiers in plant science* 12, p. 694123. DOI: 10.3389/fpls.2021.694123.

Hurtado, Grecia; Olbricht, Klaus; Mercado, Jose A.; Pose, Sara; Knoche, Moritz (2024): Phenotyping 172 strawberry genotypes for water soaking reveals a close relationship with skin water permeance. In *PeerJ* 12, e17960. DOI: 10.7717/peerj.17960.

James, C. M.; Wilson, F.; Hadonou, A. M.; Tobutt, K. R. (2003): Isolation and characterization of polymorphic microsatellites in diploid strawberry (Fragaria vesca L.) for mapping, diversity studies and clone identification. In *Molecular Ecology Notes* 3 (2), pp. 171–173. DOI: 10.1046/j.1471-8286.2003.00365.x.

Jung, Sook; Lee, Taein; Cheng, Chun-Huai; Buble, Katheryn; Zheng, Ping; Yu, Jing et al. (2019): 15 years of GDR: New data and functionality in the Genome Database for Rosaceae. In *Nucleic acids research* 47 (D1), D1137-D1145. DOI: 10.1093/nar/gky1000.

Liu, Tianjia; Li, Muzi; Liu, Zhongchi; Ai, Xiaoyan; Li, Yongping (2021): Reannotation of the cultivated strawberry genome and establishment of a strawberry genome database. In Horticulture research 8 (1), p. 41. DOI: 10.1038/s41438-021-00476-4.

Monfort, A.; Vilanova, S.; DAVIS, T. M.; Arús, P. (2006): A new set of polymorphic simple sequence repeat (SSR) markers from a wild strawberry (Fragaria vesca ) are transferable to other diploid Fragaria species and to Fragaria × ananassa. In *Molecular Ecology Notes* 6 (1), pp. 197–200. DOI: 10.1111/j.1471-8286.2005.01191.x.

Njuguna, Wambui (2010): Development and Use of Molecular Tools in Fragaria. Dissertation. Oregon State University, Oregon.

Revelle W. (2026) psych: Procedures for Psychological, Psychometric, and Personality Research. https://CRAN.R-project.org/package=psych

Zorrilla-Fontanesi, Yasmín; Cabeza, Amalia; Torres, Ana M.; Botella, Miguel A.; Valpuesta, Victoriano;

Monfort, Amparo et al. (2011): Development and bin mapping of strawberry genic-SSRs in diploid Fragaria and their transferability across the Rosoideae subfamily. In *Mol Breeding* 27 (2), pp. 137–156. DOI: 10.1007/s11032-010-9417-1.
